# Remote control of neural function by X-ray-induced scintillation

**DOI:** 10.1038/s41467-021-24717-1

**Published:** 2021-07-22

**Authors:** Takanori Matsubara, Takayuki Yanagida, Noriaki Kawaguchi, Takashi Nakano, Junichiro Yoshimoto, Maiko Sezaki, Hitoshi Takizawa, Satoshi P. Tsunoda, Shin-ichiro Horigane, Shuhei Ueda, Sayaka Takemoto-Kimura, Hideki Kandori, Akihiro Yamanaka, Takayuki Yamashita

**Affiliations:** 1 Department of Neuroscience II, Research Institute of Environmental Medicine, Nagoya University, Nagoya, Japan; 2 Department of Neural Regulation, Graduate School of Medicine, Nagoya University, Nagoya, Japan; 3 Department of Physiology, Fujita Health University School of Medicine, Toyoake, Japan; 4 Nara Institute of Science and Technology, Nara, Japan; 5 Department of Computational Biology, Fujita Health University School of Medicine, Toyoake, Japan; 6 International Research Center for Medical Sciences, Kumamoto University, Kumamoto, Japan; 7 Department of Life Science and Applied Chemistry, Nagoya Institute of Technology, Nagoya, Japan; 8 PRESTO, Japan Science and Technology Agency, Kawaguchi, Japan; 9 Department of Neuroscience I, Research Institute of Environmental Medicine, Nagoya University, Nagoya, Japan; 10 Department of Molecular/Cellular Neuroscience, Graduate School of Medicine, Nagoya University, Nagoya, Japan; 11 CREST, Japan Science and Technology Agency, Kawaguchi, Japan

**Keywords:** Behavioural methods, Optogenetics, Fear conditioning

## Abstract

Scintillators emit visible luminescence when irradiated with X-rays. Given the unlimited tissue penetration of X-rays, the employment of scintillators could enable remote optogenetic control of neural functions at any depth of the brain. Here we show that a yellow-emitting inorganic scintillator, Ce-doped Gd_3_(Al,Ga)_5_O_12_ (Ce:GAGG), can effectively activate red-shifted excitatory and inhibitory opsins, ChRmine and GtACR1, respectively. Using injectable Ce:GAGG microparticles, we successfully activated and inhibited midbrain dopamine neurons in freely moving mice by X-ray irradiation, producing bidirectional modulation of place preference behavior. Ce:GAGG microparticles are non-cytotoxic and biocompatible, allowing for chronic implantation. Pulsed X-ray irradiation at a clinical dose level is sufficient to elicit behavioral changes without reducing the number of radiosensitive cells in the brain and bone marrow. Thus, scintillator-mediated optogenetics enables minimally invasive, wireless control of cellular functions at any tissue depth in living animals, expanding X-ray applications to functional studies of biology and medicine.

## Abstract (web summary)

Scintillators emit visible luminescence when irradiated with X-rays and may enable remote optogenetic control of neurons deep in the brain. The authors inject an inorganic scintillator to activate and inhibit midbrain dopamine neurons in freely moving mice by X-ray irradiation to modulate place preference behavior.

## Introduction

Optogenetics has enabled the elucidation of the causal roles of specific neurons in driving circuit dynamics, plasticity, and behavior^1,2^. Clinical treatment of neurological diseases may also benefit from optogenetic approaches that can control functions of well-defined neural circuits in precise timings^3–7^. However, the application of optogenetics to deep brain regions usually requires the invasive implantation of optical fibers tethered to an external light source because the stimulating light (wavelength: ~430–610 nm) used to activate light-sensitive proteins is heavily scattered and absorbed by tissues^1^. These tethered fiber optics, although widely employed, are known to pose diverse problems, including tissue damage, neuroinflammatory responses, phototoxicity and thermal effects upon irradiation, as well as physical restriction of animal movement^8–11^. Recent studies have shown that injectable upconverting nano/microparticles, which emit visible light in response to tissue-penetrating near-infrared (NIR) light irradiation, can be used for minimally invasive actuation of neurons deep in the brain^12–16^. However, even NIR light penetrates only up to several millimeters of tissue. Furthermore, the low upconversion yields of these particles demand high-energy NIR illumination which can cause abrupt tissue heating and photodamage^15,16^. Non-optical forms of energy delivery to control the activities of specific neuronal populations using magnetothermal^17^ and ultrasonic^18^ stimulation have also been explored; however, these approaches are associated with a significantly reduced time resolution compared with optogenetics and are currently restricted by limited compatibility with free behavior.

Here, we report the development of an X-ray-mediated, wireless optogenetic technology, which is practically unconstrained by tissue depth. We employ a scintillator that can absorb the energy of incoming X-ray particles and release it in the form of visible luminescence called scintillation. Scintillators are widely used in various particle detectors, such as X-ray security and computed tomography (CT) scanners. The concept that scintillators could potentially serve as optogenetic actuators has already been proposed^19,20^. However, it has not yet been proven whether scintillation induced by X-rays can effectively manipulate neural functions in behaving animals by activating light-sensitive proteins. Also, it is currently unknown whether scintillators are biocompatible and can be safely implanted in living animals. We found that an inorganic scintillator, Ce-doped Gd_3_(Al,Ga)_5_O_12_ (Ce:GAGG), emits yellow scintillation^21,22^ to effectively activate red-shifted excitatory and inhibitory opsins. Using Ce:GAGG microparticles, we were able to actuate a specific neuronal population in freely moving mice, driving-related behaviors with X-ray irradiation that does not harm radiosensitive cells. Our analysis also shows the biocompatibility of Ce:GAGG microparticles. Overall, this work demonstrates that X-rays can be used to control the function of cells at any tissue depth, expanding the range of X-ray applications in biology and medicine.

## Results

### Ce:GAGG luminescence activates red-shifted opsins

**Fig. 1:**
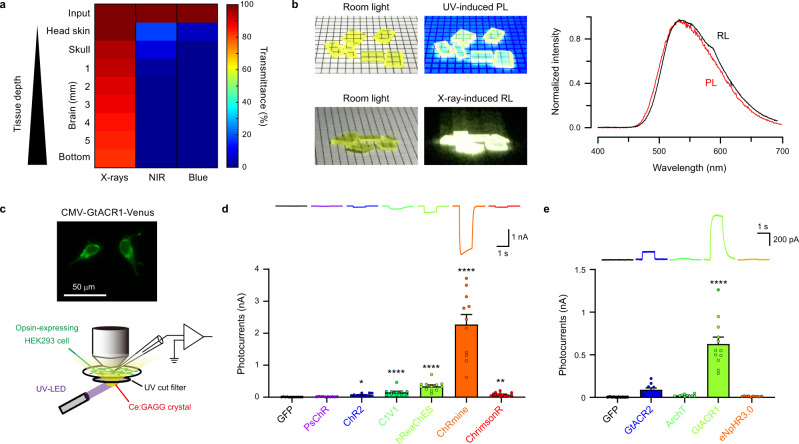
Potential of Ce:GAGG scintillation for application in deep brain optogenetics. **a** Tissue transmittance of X-rays (150 kV, 3 mA), NIR (976 nm), and blue light (470 nm) penetrating through mouse head tissues from a dorsal position. Values were estimated using the data shown in Supplementary Fig. 1. **b** Left, Ce:GAGG crystals under room light. Middle, Ce:GAGG crystals under UV (365 nm) and X-rays (150 kV, 3 mA). Right, spectra of RL (black) and PL (red) emitted by a single Ce:GAGG crystal. The luminescence intensity was normalized at the peak. Grid-scale, 2 mm. **c** Top, the epi-fluorescence image of HEK 293 cells expressing GtACR1-Venus. Similar fluorescence images were observed in 30 recording sessions (134 cells in total). Bottom, schematic of the photo-current recording. **d** Representative photocurrents (top) and photocurrent amplitudes (bottom) of different excitatory opsins and GFP induced by Ce:GAGG PL (GFP, *n* = 12 cells; PsChR, *n* = 10 cells, *p* > 0.9999; ChR2, *n* = 12 cells, *p = 0.0205; C1V1, *n* = 11 cells, ****p < 0.0001; bReaChES, *n* = 12 cells, ****p < 0.0001; ChRmine, *n* = 11 cells, ****p < 0.0001; ChrimsonR, *n* = 14 cells, **p = 0.0047; Dunn’s multiple comparison test vs. GFP, two-sided). PL power: 1.8 mW/cm^2^. **e** Same as **d** but with inhibitory opsins and GFP (GFP, *n* = 10 cells; GtACR2, *n* = 10 cells, *p* = 0.440; ArChT, *n* = 10 cells, *p* = 0.994; GtACR1, *n* = 12 cells, ****p < 0.0001; eNpHR3.0, *n* = 10 cells, *p* = 0.9998; Dunnett’s multiple compariso*n* test vs. GFP, two-sided). PL power: 1.8 mW/cm^2^. Circles in **d** and **e** indicate individual cells. Values are mean ± SEM.

When irradiated onto a mouse head (Fig. 1a and Supplementary Fig. 1), X-rays readily penetrated through the head skin, skull, and brain tissue, whereas most of the energy derived from NIR and visible light did not reach deep into the brain due to absorption and scattering by tissue. Moreover, X-ray irradiation did not increase the temperature of tissues, whereas NIR illumination with a conventional optogenetic stimulation protocol caused striking tissue heating (Supplementary Fig. 2). These results highlight the distinct advantages of using X-ray-induced scintillation for remote optogenetic control of neural circuits deep in the brain. In this study, we utilized single scintillator crystals of Ce:GAGG which emit yellow luminescence in response to UV or X-ray radiation^21,22^ (Fig. 1b). These crystals were transparent and non-deliquescent (Fig. 1b). UV-induced photo-luminescence (PL) and X-ray-induced radio-luminescence (RL) of a Ce:GAGG crystal has essentially the same spectrum^21,22^ (peak wavelength: 520–530 nm; Fig. 1b) because both PL and RL are based on the 5d-4f transitions of Ce^3+^. The RL light yield of Ce:GAGG is reported to be 46,000 photons/MeV^21,22^.

We first sought opsins that could be effectively activated by the PL of Ce:GAGG. DNA plasmids encoding different opsins were transfected into HEK 293 cells and photocurrents induced by PL illumination were measured (Fig. 1c). For depolarizing opsins, the yellow PL of Ce:GAGG (1.8 mW/cm^2^) elicited the largest photocurrents in cells expressing the red-shifted opsin ChRmine^23^ (2.28 ± 0.31 nA, *n* = 11; Fig. 1d). Regarding inhibitory opsins, the anion channelrhodopsin GtACR1^24^ showed the strongest activation (627.2 ± 80.2 pA, *n* = 12; Fig. 1e) in response to the PL of Ce:GAGG. PL-irradiation of GFP-expressing cells induced undetectable currents (Fig. 1d, e). Thus, Ce:GAGG PL can activate red-shifted opsins that are used for optogenetic control of neurons. Furthermore, the yellow PL of Ce:GAGG could also induce significant activation of the enzyme rhodopsin BeGC1^25^ (Supplementary Fig. 3), suggesting that the scintillation of Ce:GAGG could be used for the activation of various light-sensitive proteins.

### Bidirectional control of neuronal activities by Ce:GAGG luminescence

**Fig. 2:**
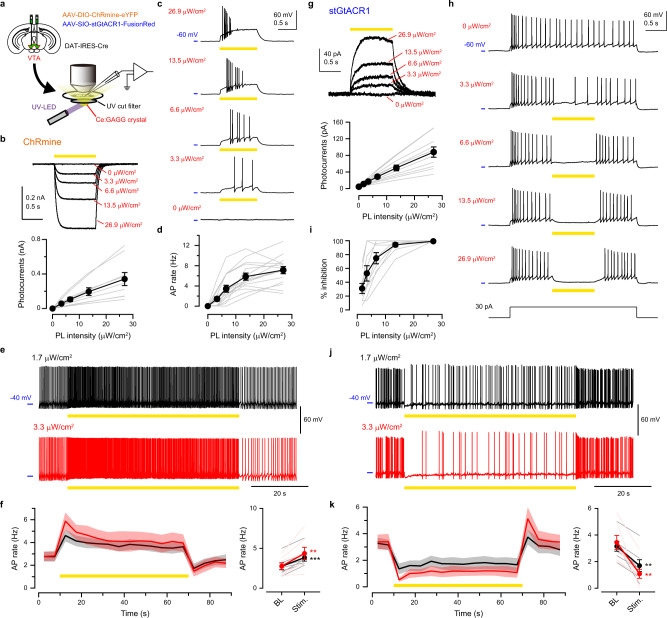
Photo-luminescence of Ce:GAGG bidirectionally actuated VTA-DA neurons in vitro. **a** Schematic of the experiment. **b** Top, sample voltage-clamp recordings from a ChRmine-expressing VTA-DA neuron responding to 1-s Ce:GAGG PL. Bottom, photocurrent amplitude vs. PL intensity (*n* = 9 cells). **c** Sample recordings from a ChRmine-expressing VTA-DA neuron current-clamped at approximately −60 mV, responding to 1-s PL. **d** AP rate of ChRmine-expressing neurons vs. PL intensity (*n* = 15 cells). **e** Sample recordings from a ChRmine-expressing neuron current-clamped at approximately −40 mV, responding to 1-min PL illumination. **f** Time course (left) and quantification (right) of average AP rates with 1-min PL illumination at 1.7 (black) or 3.3 (red) μW/cm^2^. Thick line: mean, Shadows: ± SEM, BL: baseline, Stim.: stimulation. 1.7 μW/cm^2^: *n* = 7 cells, ***p = 0.0002; 3.3 μW/cm^2^: *n* = 10 cells, **p = 0.0017; paired *t*-test, two-sided. **g** Same as **b**, but from stGtACR1-expressing VTA-DA neurons (*n* = 11 cells). **h** Same as **c**, but for a stGtACR1-expressing neuron. APs were evoked by the current injection. **i** Success rate of spike suppression by Ce:GAGG PL in stGtACR1-expressing neurons (*n* = 10 cells). **j**, **k** Same as **e**, **f** but for stGtACR1-expressing neurons. 1.7 μW/cm^2^: *n* = 7 cells, **p = 0.0017; 3.3 μW/cm^2^: *n* = 7 cells, **p = 0.0034; paired *t*-test, two-sided. Lightly colored lines indicate individual cells. Values are mean ± SEM.

We next examined the intensity of Ce:GAGG luminescence required to actuate neuronal activities. Cre-dependent adeno-associated virus (AAV) vectors were injected into the ventral tegmental area (VTA) of DAT-IRES-Cre mice to induce the specific expression of ChRmine in dopamine (DA) neurons (Fig. 2a and Supplementary Fig. 4a, b). In acute slice preparations, 1-s pulses of the yellow PL of Ce:GAGG elicited depolarizing photocurrents in DA neurons in a PL intensity-dependent manner (Fig. 2b). Irradiation with 3.3 μW/cm^2^ elicited action potentials (APs) in 10 out of 15 DA neurons which were current-clamped to −60 mV, and the rate of PL-evoked APs plateaued at approximately 15 μW/cm^2^ (Fig. 2c, d). When the cells were held at around −40 mV to exhibit spontaneous APs, 1-min PL illumination (Fig. 2e, f), and 1-s pulsed PL illumination (5–20 Hz; Supplementary Fig. 5) increased the AP rate at an even lower intensity (1.7 μW/cm^2^). Essentially the same results were obtained in ChRmine-expressing neurons of the medial septum (Supplementary Fig. 6). In VTA-DA neurons expressing inhibitory soma-targeted GtACR1^26^ (stGtACR1; Supplementary Fig. 4c, d), hyperpolarizing photocurrents were induced by Ce:GAGG PL illumination (Fig. 2g), which attenuated AP generation at 1.7 μW/cm^2^ and maximally suppressed spiking at around 15 μW/cm^2^ PL (Fig. 2h–k). Thus, the activity of ChRmine-expressing or stGtACR1-expressing neurons can be modulated by illumination with Ce:GAGG PL at intensities of a few microwatts.

### Ce:GAGG microparticles enable neuronal actuation in vivo

**Fig. 3:**
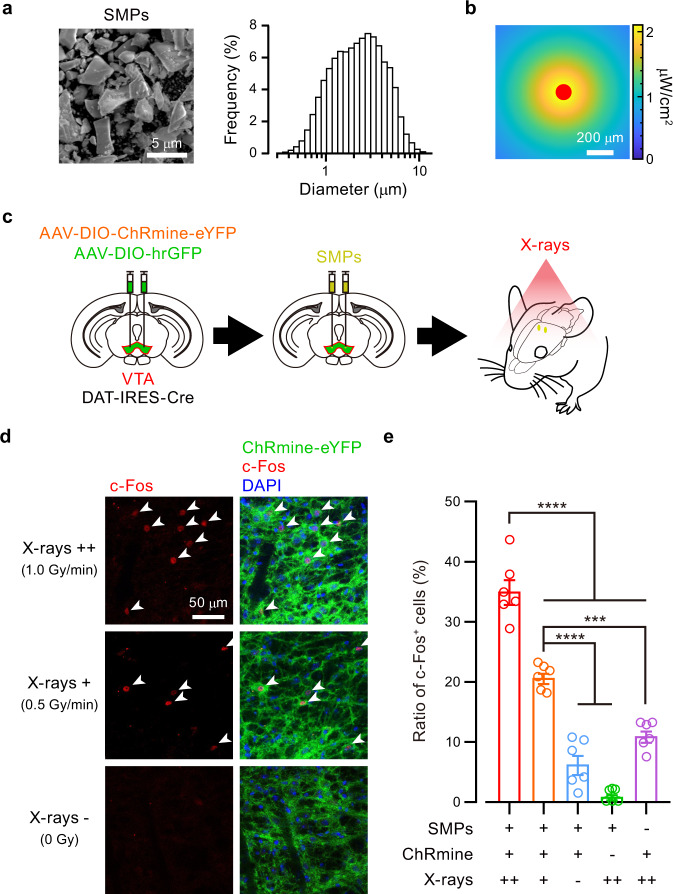
Radio-luminescence of Ce:GAGG microparticles activated VTA-DA neurons in vivo. **a** Left, a scanning electron micrograph of SMPs. Right, the size distribution of the SMPs measured by dynamic light scattering. **b** An RL intensity map around the SMPs (red) injected in the gray matter at a depth of 4.2 mm under 1.0 Gy/min X-irradiation (see also Supplementary Fig. 7). **c** Schematic of the c-Fos induction experiment. **d** Representative confocal images showing RL-induced expression of c-Fos (red, arrowheads) among ChRmine-expressing cells near injected SMPs. **e** Ratio of c-Fos-positive cells under different conditions (*n* = 6 hemispheres from 3 mice for each group). SMPs+/ChRmine+/X-rays++ vs. SMPs+/ChRmine+/X-rays+, ****p < 0.0001; SMPs+/ChRmine+/X-rays++ vs. SMPs+/ChRmine+/X-rays–, ****p < 0.0001; SMPs+/ChRmine+/X-rays++ vs. SMPs+/ChRmine–/X-rays++, ****p < 0.0001; SMPs+/ChRmine+/X-rays++ vs. SMPs–/ChRmine+/X-rays++, ****p < 0.0001; SMPs+/ChRmine+/X-rays+ vs. SMPs+/ChRmine+/X-rays–, ****p < 0.0001; SMPs+/ChRmine+/X-rays+ vs. SMPs+/ChRmine–/X-rays++, ****p < 0.0001; SMPs+/ChRmine+/X-rays+ vs. SMPs–/ChRmine+/X-rays++, ***p = 0.0002; Bonferroni’s multiple comparison test, two-sided. Open circles indicate individual data. Values are mean ± SEM.

Having identified scintillator-opsin combinations that modulate neuronal activity, we next examined the ability of X-ray-induced RL of the Ce:GAGG crystal to activate neurons in vivo. We first pulverized the Ce:GAGG crystal into particles with an average size of 2.3 μm (named scintillator microparticles, SMPs; Fig. 3a), to be injected into the brain. The SMPs injected in vivo (50 mg/ml, 600 nl) formed clusters with an average diameter of 119 ± 13 μm (*n* = 12 mice). The intensity of RL emitted by SMPs, which was proportional to the dose rate of X-irradiation (Supplementary Fig. 7), can reach ~2 μW/cm^2^ near the injection site at the VTA with X-irradiation onto the mouse head at a rate of 1.0 Gy/min (Fig. 3b). We next induced the expression of ChRmine in VTA-DA neurons using AAV injections and injected the SMPs (50 mg/ml, 600 nl) at the same location as the AAV injection (Fig. 3c). X-irradiation for a total of 5 min (1-min pulses every 2 min, five times, at 0.5 or 1.0 Gy/min) induced c-Fos expression in a larger fraction of ChRmine-expressing neurons compared to control conditions (Fig. 3d, e and Supplementary Fig. 8). The fraction of c-Fos-positive cells increased as the dose rate of X-irradiation increased. Thus, the SMPs injected in vivo can activate neurons in an X-ray-dependent and opsin-dependent manner.

**Fig. 4:**
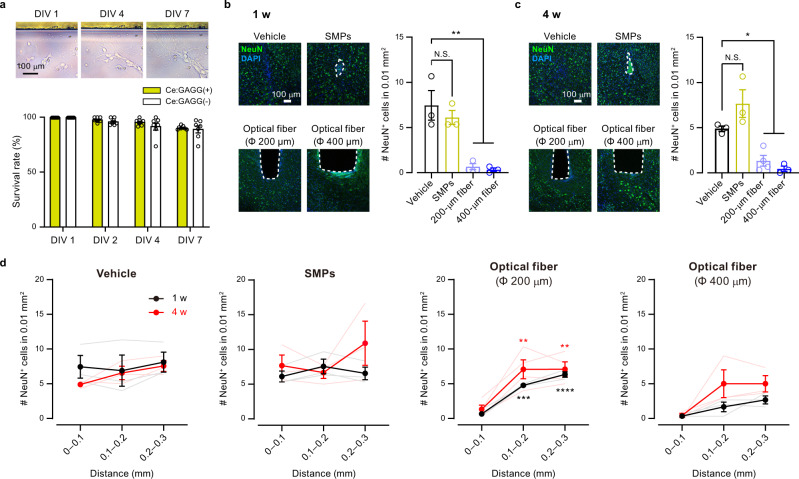
Ce:GAGG microparticles were non-cytotoxic and biocompatible. **a** Top, dissociated hippocampal neurons cultured with a Ce:GAGG crystal (upper). Bottom, the survival rates of dissociated neurons cultured with or without a Ce:GAGG crystal (*n* = 7 dishes for each group, *F*_(1,24)_ = 2.18, *p* = 0.15; two-way ANOVA). **b**, **c** Left, representative confocal images of immunoreactivity against NeuN (green) at the injection site of vehicle or SMPs, and at the ventral tip of an implanted optical fiber with a diameter of 200 μm or 400 μm, at one week (1 w) (**b**) or four weeks (4 w) (**c**) after surgery. The trace of SMPs or optical fiber is outlined by a dashed line. Blue: DAPI. Right, an average number of NeuN-positive cells counted in a 100 μm × 100 μm square near the injection/implantation traces. 1 w: Vehicle, *n* = 3 mice; SMPs, *n* = 3 mice, *p* = 0.633, *n* = 3 mice, 200-μm fiber, *n* = 3 mice, **p = 0.0022; 400-μm fiber, *n* = 3 mice, **p = 0.0016. 4 w: Vehicle, *n* = 3 mice, SMPs, *n* = 3 mice, *p* = 0.106; 200-μm fiber, *n* = 4 mice, *p = 0.0277; 400-μm fiber, *n* = 3 mice, *p = 0.0121; Dunnett’s multiple comparison test vs. the vehicle group, two-sided. Neuronal loss was found near the optical fiber traces, but not around implanted SMPs. **d** Mean number of NeuN-positive cells in a 100 μm × 100 μm square at different distances from the injection/implantation traces at 1 w or 4 w after surgery. 1 w: *n* = 3 mice for each group; 4 w: Vehicle, *n* = 3 mice, SMPs, *n* = 3 mice, 200-μm fiber, *n* = 4 mice, 400-μm fiber, *n* = 3 mice. 1 w-Vehicle, *F*_(2,6)_ = 0.114, *p* = 0.894; SMPs, *F*_(2,6)_ = 0.643, *p* = 0.559; 200-μm fiber, *F*_(2,6)_ = 4.7, *p* < 0.0001; 400-μm fiber, *F*_(2,6)_ = 4.83, *p* = 0.0563; one-way ANOVA. 4 w-Vehicle, *F*_(2,6)_ = 3.34, *p* = 0.106; SMPs, *F*_(2,6)_ = 1.11, *p* = 0.390; 200-μm fiber, *F*_(2,9)_ = 9.88, *p* = 0.0054; 400-μm fiber, *F*_(2,6)_ = 3.80, *p* = 0.0857; one-way ANOVA. 200-μm fiber: 1 w, 0.1–0.2 mm, ***p = 0.0001; 1 w, 0.2–0.3 mm, ****p < 0.0001; 4 w, 0.1–0.2 mm, **p = 0.0072; 4w, 0.2–0.3 mm, **p = 0.0072; Dunnett’s multiple comparison test vs. the 0–0.1 mm group, two-sided. Open circles and lightly colored lines indicate individual data. N.S., not significant. Values are mean ± SEM.

We further assessed the biocompatibility of the scintillator. The vast majority of dissociated hippocampal neurons around the Ce:GAGG crystal in the culture dish survived for 7 days (Fig. 4a). Similarly, HEK 293 cells cultured in the presence of the Ce:GAGG crystal proliferated at a normal rate (Supplementary Fig. 9). Furthermore, SMPs implanted into the brain for one week caused less activation of microglial cells than that caused by implantation of an optical fiber (Supplementary Fig. 10). Notably, the number of neurons was significantly reduced at the immediate surrounding (<100 μm) of the implanted optic fibers (diameter, 200 μm; one week after surgery; 9.0% of control, *p* = 0.0022; four weeks after surgery, 27.3% of control, *p* = 0.027; Fig. 4b–d), whereas that was unchanged around injected SMPs (one week after surgery, 82.1% of control, *p* = 0.63; four weeks after surgery, 157% of control, *p* = 0.10; Fig. 4b–d). The clusters of the injected SMPs did not change in size even after a long period of implantation (Supplementary Fig. 10c). Furthermore, the weight of rod-shaped Ce:GAGG crystals (size: 0.5 mm × 0.5 mm × 1.0–1.5 mm) implanted in the brain did not change four weeks after implantation (Supplementary Fig. 10d), suggesting that Ce:GAGG crystals may not be degraded in the tissue for a long period. Thus, the Ce:GAGG crystals have excellent biocompatibility, and the SMPs can stay at the injection site for a long period without causing cytotoxicity.

### Bidirectional change of behaviors induced by scintillator-mediated optogenetics

**Fig. 5:**
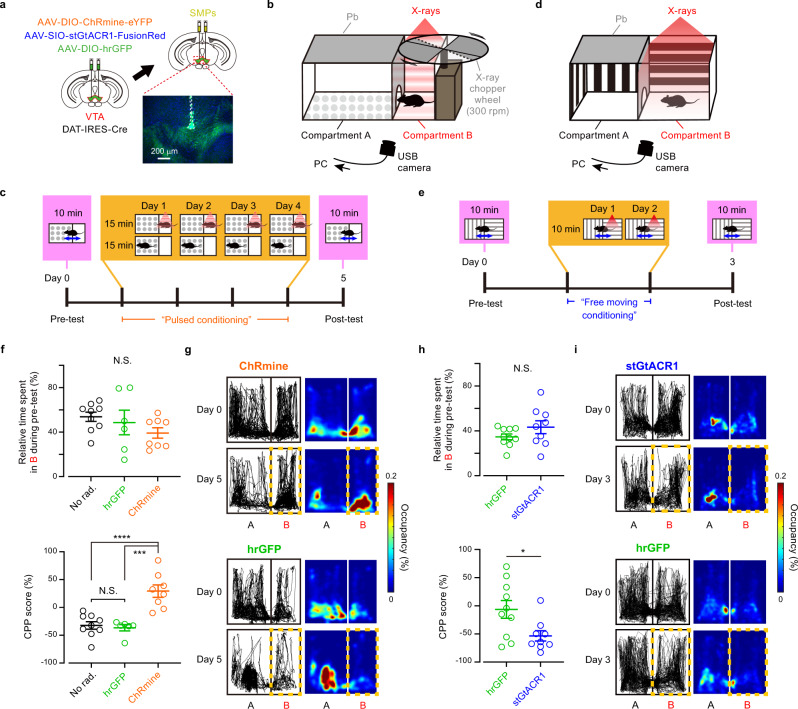
Scintillator-mediated wireless optogenetics drove conditioned place preference and aversion in freely behaving mice. **a** Schematic of the experiment. Inset: an epi-fluorescence image showing SMPs (dashed outline) injected at dorsal VTA. Green: ChRmine-eYFP, Blue: DAPI. Similar results were obtained in 21 mice. **b** CPP test chamber for “Pulsed conditioning” (PC). The X-ray shieldings and the X-ray wheel chopper are made of lead (Pb). **c** Time course of CPP test with PC. The gate between two compartments is open for pre-tests and post-tests but closed during PC. **d** CPP test chamber for “Free moving conditioning” (FC). **e** Time course of CPP test with FC. The gate between the two compartments is open throughout the test. **f** Quantification of CPP with PC (*n* = 9 mice for no radiation control [No rad.] group, 6 mice for hGFP group, 8 mice for ChRmine group). Pre-test (top), *F*_(2,20)_ = 1.48, *p* = 0.252; post-test (bottom), *F*_(2,20)_ = 19.0, *p* < 0.0001; one-way ANOVA. Post-test (bottom): No rad. vs. hrGFP, *p* > 0.9999; No rad. vs. ChRmine, ****p < 0.0001; hrGFP vs. ChRmine, ***p = 0.0001; Bonferroni’s multiple comparison test, two-sided. **g** Representative tracking data (left) and the corresponding heat maps (right) for mice before (Day 0) and after (Day 5) PC, as extracted from oblique-view movies. **h** Quantification of CPP with FC (*n* = 10 mice for hGFP group, 9 mice for stGtACR1 group). Pre-test (top), *p* = 0.243; post-test (bottom), *p = 0.0435; Mann–Whitney U test, two-sided. **i** Same as **g**, but for mice before (Day 0) and after (Day 3) FC. N.S., not significant. Open circles indicate individual data. Values are mean ± SEM.

We finally tested whether the scintillator-mediated actuation of neurons in vivo could induce behavioral changes. Transient activation and inhibition of DA neurons in the VTA are sufficient for behavioral conditioning^27–29^. We, therefore, induced the expression of excitatory ChRmine or inhibitory stGtACR1 in VTA-DA neurons through viral injections and bilaterally injected SMPs in the VTA (Fig. 5a). The conditioned place preference (CPP) test was performed by placing the mice into a test chamber with two compartments, only one of which was irradiated with either X-ray pulses (50 ms, 10 Hz, 10 times every 30 s) through an X-ray chopper wheel (“Pulsed conditioning” [PC]; Fig. 5b, c and Supplementary Fig. 11) or continuous X-ray irradiation (“Free moving conditioning” [FC]; Fig. 5d, e and Supplementary Fig. 11). The initial place preference was not different between the opsin-expressing and GFP-expressing control mice. After PC, however, mice expressing ChRmine had a significantly higher preference for the X-ray conditioned compartment than control mice (Fig. 5f, g), whereas those expressing stGtACR1 had a lower preference for the conditioned compartment after FC (Fig. 5h, i). The CPP score of GFP-expressing mice was similar to that of control mice conditioned without X-irradiation (Fig. 5f), suggesting that X-irradiation itself has no effect on place preference. Thus, scintillator-mediated remote optogenetics can be used for bidirectional neuronal actuation deep in the brain of mice, resulting in behavioral changes.

**Fig. 6:**
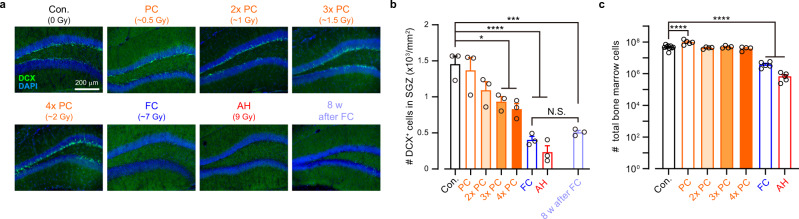
X-ray dose-dependent effects on immature neurons and bone marrow cells. **a** Representative epi-fluorescence images of doublecortin (DCX) immunoreactivity (green) at the hippocampal dentate gyrus two days after acute high-dose radiation (AH), “Pulsed conditioning” (PC), double (2×) PC, triple (3×) PC, quadruple (4×) PC, “Free moving conditioning” (FC), or no radiation (Con), or at 8 weeks after FC radiation (8 w after FC). Blue: DAPI. **b** Quantification of DCX-positive cells under different conditions (*n* = 3 mice for each group). Con vs. PC, *p* > 0.9999; Con vs. 2× PC, *p* = 0.531; Con vs. 3× PC, *p = 0.0488; Con vs. 4× PC, *p = 0.0102; Con vs. FC, ****p < 0.0001; Con vs. AH, ****p < 0.0001; Con vs. 8 w after FC, ***p = 0.0001; FC vs. 8 w after FC, *p* > 0.9999; Bonferroni’s multiple comparison test, two-sided. **c** Quantification of total bone marrow cells three days after X-irradiation (Con, *n* = 9 mice; PC, *n* = 5 mice; 2× PC; *n* = 4 mice; 3× PC, *n* = 4 mice; 4× PC, *n* = 4 mice; FC, *n* = 5 mice; AH, *n* = 5 mice). PC, ****p < 0.0001; 2× PC, *p* = 0.987; 3× PC, *p* = 0.9998; 4× PC, *p* = 0.887; FC, ****p < 0.0001; AH, ****p < 0.0001; Dunnett’s multiple com*p*arison tests vs. Con, two-sided. N.S.: not significant. Open circles indicate individual data. Values are mean ± SEM.

The total dose of X-irradiation during the behavioral tests is far below the threshold for acute neuronal and vascular dysfunction in the brain^30,31^. X-irradiation corresponding to the FC (~7 Gy) did not change locomotor behavior (Supplementary Fig. 12) and blood–brain barrier function (Supplementary Fig. 13). In a long-term observation, all the mice that experienced the FC survived for at least 8 weeks after radiation with no significant difference in body weight from non-X-irradiated control mice at the same age (Supplementary Fig. 14a). These results suggest that scintillator-mediated optogenetics can be used for days-long behavioral experiments in mice even with a relatively high dose of radiation. On the other hand, the total body X-irradiation is known to damage radiosensitive cell populations, depending on its cumulative dose. A high-dose X-irradiation reduced the number of immature neurons in the hippocampal dentate gyrus (Fig. 6a, b) through apoptotic cell death of neuronal precursor cells^32,33^ (Supplementary Fig. 15). Consistent with previous reports^32,33^, hippocampal neurogenesis was partially impaired, and the number of immature neurons was not recovered at 8 weeks after the FC radiation (Fig. 6a, b; Supplementary Fig. 14b–f). However, the PC, which causes considerably less radiation dose (~0.5 Gy), induced neither loss of immature neurons (Fig. 6a, b) nor apoptosis (Supplementary Fig. 15) in the hippocampus. Although a high-dose X-irradiation caused an overall reduction in the number of bone marrow cells^34^ (Fig. 6c) without specificity of cell-types (Supplementary Fig. 16), the PC rather increased the number of the total bone marrow cells (Fig. 6c) presumably due to radio-resistance effects^35^. Increasing the total radiation dose to ~1.5 Gy (three times the PC dose) significantly reduced the number of immature neurons in the hippocampus (Fig. 6a, b), whereas four times (~2 Gy) or less PC did not reduce the number of total bone marrow cells (Fig. 6c). Thus, reducing the radiation dose to less than two times of PC will allow for the safer application of scintillator-mediated optogenetics.

## Discussion

We have here demonstrated the feasibility of a scintillator-mediated optogenetic technology that allows full wireless control of neuronal activity in behaving animals. Our electrophysiological recordings (Fig. 2; Supplementary Figs. 5 and 6), c-Fos-induction experiments (Fig. 3), and behavioral experiments (Fig. 5) collectively suggest that scintillation of Ce:GAGG particles could bi-directionally manipulate the activities of opsin-expressing neurons in vivo by X-irradiation. The scintillator particles were biocompatible and injectable and remained at the injection site for long periods without notable cytotoxicity (Fig. 4), serving as minimally invasive optogenetic actuators. The thermal effects on neuronal activity were negligible using this technology (Supplementary Fig. 2), a significant advantage over conventional^9,11^ and NIR-mediated^15,16^ optogenetics. Besides, scintillator-mediated optogenetics does not require chronic implantation of large devices such as power modules^8,36,37^, which would be another advantage over existing LED-based wireless optogenetic methods. Using a careful radiation dose setting, this technology can be safely applied in a variety of rodent behavioral experiments such as those with more naturalistic, context-rich settings, which are normally hindered by the tethered fiber optics, or the large implant on the head in other wireless optogenetic technologies^8,36^. By employing flexible biomaterials containing scintillator particles, it might be possible to control nerve activities in the spinal cord and peripheral nervous system of freely moving rodents with less invasive procedures than practiced in conventional^37,38^ or NIR-mediated^39^ optogenetics. Given the unlimited tissue penetration of X-rays, scintillator-mediated optogenetics may also be applied to larger animals, including monkeys.

Scintillator-mediated optogenetics would also be advantageous for a combination of neuronal manipulations with electrophysiology or imaging because tethered optic fibers or implanted devices often limit the physical space for simultaneous recording. For example, large-scale Ca^2+^ imaging from the cerebral cortex in head-restrained^40^ or freely moving^41,42^ rodents can be combined with X-ray-mediated, wireless manipulation of the activities of specific neurons in subcortical structures, which is otherwise practically impossible. In chronic electrophysiology or endoscope imaging, the implantation of a microdrive or a miniature microscope on the skull makes it difficult to be combined with optogenetics apart from photo-stimulation around the recording site. However, with scintillator-mediated optogenetics, it would be much easier to manipulate neuronal activities at any target region by injecting AAV and SMPs before implantation.

The total radiation dose of our PC procedure (~0.5 Gy), which is roughly equivalent to the dose of a single perfusion CT scan^43,44^, is more than 100 times lower than the standard dose of radiotherapy for brain tumor treatment (50–70 Gy^30,31^). Our experiments on radiation toxicity revealed that the maximal radiation dose for safer experiments using free moving mice would be around 1 Gy. This dose level corresponds to 2400 pulses of 50 ms optogenetic stimuli, which enables repetitive interrogations of neural functions in single animals. Blockade of neuroinflammation is known to ameliorate impairment of neurogenesis caused by radiation^45^, which may increase the applicability of our technology for longer-term experiments. Although we have shown that scintillator-mediated optogenetics can be applied to behavioral experiments at a non-toxic cumulative dose, increased safety in the use of this technology can be achieved by focal X-irradiation of the brain, which prevents radiation exposure to other organs. In experiments using head-restrained animals, simple shielding would enable such focal X-irradiation. Stereotactic focusing of small radiation beams on specific brain regions, as was achieved in gamma knife surgery^46^, may provide the further safer application of this technology.

Since Röntgen’s discovery in the late 19th century^47^, X-rays have been widely used for medical imaging and cancer therapy. However, X-rays have never been used to control the physiological functions of cells in living animals, as we have shown here. The development of scintillator-mediated optogenetics thus expands the application of X-rays to functional studies of biology and medicine. Biomedical technologies that use visible light for genome editing^48^ or control of intracellular signaling^49^ would benefit from wireless applications targeting deeper tissues, which is now possible with scintillator-mediated approaches.

There have been many potential candidates for scintillator materials that could be used in X-ray-based optogenetics^19,20^. The Ce:GAGG crystal has been one of the scintillators with the highest light yield^21,22^. However, the Ce:GAGG crystal is also a fluorescent material with a relatively broad emission/excitation spectrum^22^, which hampers simultaneous imaging and neuronal manipulations in the same neuronal populations. Another limitation of the current technique is that the intensity of the RL emitted by Ce:GAGG microparticles in vivo is not high enough to instantaneously induce action potentials in neurons with millisecond temporal precision. Clearly, our study is therefore only a first step toward establishing safe and efficient X-ray-based optogenetics for controlling cellular functions. This study demonstrates the feasibility of the use of X-rays for functional studies, providing evidence for bi-directional modulation of neural functions in behaving animals by X-ray irradiation. Future improvements in light yields of scintillators, engineering of opsin-bound scintillator nanocrystals, and combination with focused X-irradiation will all contribute to allowing control of cellular functions over larger volumes of tissue with less risk of radiation toxicity.

## Methods

### Scintillator preparation

The single crystal Ce:GAGG was synthesized using the conventional Czochralski method^21,22^. For electrophysiology (Figs. 1 and 2, and Supplementary Figs. 5 and 6) and other measurements (Supplementary Figs. 2, 3, and 9), the crystal was fabricated into 3 − 8 mm rectangular blocks 0.5 − 1 mm thick. For injection of the crystals in the mouse brain, we pulverized the Ce:GAGG crystals into particles using a planetary ball mill. These particles were further crushed in an agate mortar and collected in ethanol. The ethanol solution containing the particles was sonicated for 10 min, and smaller particles were obtained from the supernatant after removal of the precipitate. The solution was then centrifuged at 15,871×*g* for 30 s, and the supernatant was discarded. Ethanol was added to the precipitate containing Ce:GAGG particles. We repeated the sonication of the solution, the removal of precipitate, and the centrifugation to remove the supernatant a total of three times. The final precipitate contained Ce:GAGG microparticles (SMPs). After evaporation of the remaining vehicle, SMPs (50 mg/ml) were dispersed with Ringer’s solution containing (in mM) 135 NaCl, 5 KCl, 5 HEPES, 1.8 CaCl_2_, 1 MgCl_2_ (adjusted to pH 7.3 with NaOH). Rod-shaped Ce:GAGG crystals (0.5 mm × 0.5 mm × 1.0 − 1.5 mm; Supplementary Fig. 10d) were fabricated using a laser cutter and then slightly filed to round off the corners.

### Luminescence measurements

The PL of a Ce:GAGG crystal was induced with 365-nm UV light (LEDMOD V2, Omicron; LC-L2, Hamamatsu) unless otherwise noted. The PL intensity for electrophysiological recordings was routinely measured with a photodiode sensor (1 cm × 1 cm; PD300-1W, Ophir) placed over the recording chamber through a UV-cut filter fabricated from UV-cut goggles (SSUV 297, AS ONE) on each experimental day. In experiments using acute slices, the PL intensity over the slice in the recording chamber was measured. The intensity of unfiltered UV light over the recording chamber was <0.1 μW/cm^2^. The RL power of SMPs (Supplementary Fig. 7) was measured with a fiber-coupled photoreceiver (Newport 2151) through an optical fiber (tip diameter: 400 μm) placed close to a mass of SMPs exposed to X-ray irradiation in an X-ray machine (MX-160Labo, mediXtec). The photoreceiver current, which was sampled using an analog-digital converter (Picoscope 4262, Pico Technology), was calibrated against the PL power measured by the photodiode sensor and converted to luminescence intensity (in watts). For 3D simulation of the RL intensity (Fig. 3b), we assumed that the average RL intensity at the surface of a spherical aggregate of the SMPs corresponds to the measured RL power (Supplementary Fig. 7). PL emission spectra under UV illumination (340 ± 10 nm, LAX-103, Asahi Spectra) were measured using a spectrometer (QE-Pro, Ocean Optics). RL emission spectra under X-ray irradiation (70 kV, 1 mA) were measured through an optical fiber using a CCD spectrometer (DU-420-BU2, Andor)^22^.

### Animals

All experiments were performed in accordance with the guidelines of the Physiological Society of Japan and approved by the institutional review board of the Research Institute of Environmental Medicine, Nagoya University, Japan, or by the Institutional Animal Care and Use Committee of Fujita Health University, Japan. Adult C57BL6/J mice and DAT-IRES-Cre mice (B6.SJL-Slc6a3tm1.1(cre)Bkmn/J, The Jackson Laboratory) of both sexes were maintained on a 12/12-h light/dark cycle with controlled humidity (50 ± 10%) and temperature (23 ± 2 °C). Mice had free access to food and water. DAT-IRES-Cre mice were maintained as homogenic mutants (For PCR primers, see Supplementary Table 1). Only 11 − 18-week-old male DAT-IRES-Cre mice were used for the CPP experiments.

### Plasmids

For expression in HEK293 cells, all plasmids encoding opsins and fluorescent proteins were constructed by subcloning into an empty pCMV vector unless otherwise noted. pCMV-PsChR-Venus and pCMV-C1V1-Venus were obtained from H. Yawo (Tohoku University). *bReaChES-TS-eYFP* and *ChRmine-eYFP* were isolated from pAAV-CaMKIIa-DIO-bReaChes-TS-eYFP and pAAV-CaMKIIa-DIO-ChRmine-eYFP, respectively, both of which were gifted by K. Deisseroth (Stanford University). A full-length gene encoding BeGC1 (accession number KF309499) was synthesized after human codon optimization and inserted into the peGFP-N1 vector. For AAV production, pAAV-Ef1a-DIO-ChRmine-eYFP-WPRE was obtained from K. Deisseroth (Stanford University) and pAAV-hSyn1-SIO-stGtACR1-FusionRed was obtained from Addgene (#105678).

### Cell culture and transfection

For electrophysiological recordings in cultured cells, expression vector plasmids encoding opsins or hrGFP were transfected into HEK 293 cells using Lipofectamine 2000 (Thermo Fisher Scientific). The cells were washed with phosphate-buffered saline (PBS) 3–4 h after transfection and then seeded on a coverslip (12 mm diameter) in Dulbecco’s modified Eagle’s medium (DMEM; Sigma-Aldrich) supplemented with 10% (vol/vol) fetal bovine serum (FBS), 100 U/ml penicillin, and 0.1 mg/ml streptomycin. The cells were maintained in the medium in an incubator at 37 °C and 5% CO_2_/95% air for 24–36 h before recordings.

Dissociated hippocampal neurons were prepared from embryonic (E17.5) mice. Isolated hippocampal tissues were incubated with Hank’s Balanced Salt Solution (HBSS; Sigma-Aldrich) containing 1% DNase I (Sigma-Aldrich) and 2.5% trypsin for 10 min at 37 °C and then washed three times with HBSS. The tissues were then dispersed by pipetting in a Neurobasal medium (Thermo Fisher Scientific). After removing aggregated cells by filtration, the hippocampal cells were seeded on a coverslip (12 mm diameter) coated with poly-L-lysine in DMEM (Sigma-Aldrich) and incubated for 4 h at 37 °C. The culture medium was subsequently replaced by Neurobasal medium supplemented with 0.5 mM GlutaMAX (Thermo Fisher Scientific), 2% (vol/vol) B-27 (Thermo Fisher Scientific), 100 U/ml penicillin, and 0.1 mg/ml streptomycin. The cultured neurons were maintained in the medium in an incubator at 34 °C with 5% CO_2_ and 95% air.

### Viral production

For AAV production, HEK293 cells were transfected with vector plasmids including pAAV encoding an opsin, pHelper, and pAAV-RC (serotype 9 or DJ), using a standard calcium phosphate method. After three days, transfected cells were collected and suspended in lysis buffer (150 mM NaCl, 20 mM Tris pH 8.0). After four freeze-thaw cycles, the cell lysate was treated with 250 U/ml benzonase nuclease (Merck) and 1 mM MgCl_2_ for 10 − 15 min at 37 °C and centrifuged at 1753×*g* for 20 min at 4 °C. AAV was then purified from the supernatant by iodixanol gradient ultracentrifugation. The purified AAV solution was concentrated in PBS via filtration and stored at −80 °C.

### GloSensor assay

HEK293 cells were cultured in Eagle’s minimal essential medium containing L-glutamine and phenol red (Wako) supplemented with 10% (vol/vol) FBS and penicillin-streptomycin. The cells were co-transfected with the BeGC1 plasmid and the pGloSensor-42F cGMP vector (Promega) using Lipofectamine 2000 (Thermo Fischer Scientific). After transfection, 0.5 μM all-*trans*-retinal (Toronto Research Chemicals) was added to the culture medium. Before measurements, the culture medium was replaced with a CO_2_-independent medium containing 10% (vol/vol) FBS and 2% (vol/vol) GloSensor cGMP stock solution (Promega). The cells were then incubated for 2 h at 27 °C in the dark. Intracellular cGMP levels were measured by monitoring luminescence intensity using a microplate reader (Corona Electric) at 27 °C^50^.

### Stereotactic surgery

AAV-Ef1a-DIO-ChRmine-eYFP (titer: 1.6 × 10^13^ copies/ml), AAV-hSyn1-SIO-stGtACR1-FusionRed (titer: 1.1 × 10^13^ copies/ml), AAV-CMV-ChRmine-eYFP (titer: 1.2 × 10^9^ copies/ml) or AAV-CMV-DIO-hrGFP (titer: 1.5 × 10^13^ copies/ml) was injected bilaterally into the VTA (AP: from −3.0 to −3.3 mm, LM: ± 0.5 mm, depth: 4.2 mm) of DAT-IRES-Cre mice or the ventral part of the medial septum (MS; AP: 1.2 mm, LM: 0 mm, depth: 4.8 mm) of wild-type C57BL6/J mice under ~1.2% isoflurane anesthesia. Injection volume was 200 nl (VTA) or 200–400 nl (MS) per site. The mice were kept in their home cages for at least 3 weeks after AAV injection, prior to behavioral or electrophysiological experiments. For injection of SMPs, SMPs (50 mg/ml) were dispersed with Ringer’s solution. The SMPs were bilaterally injected into the VTA with a volume of 600 nl per site at the same coordinates as AAV injections. In some mice, the SMPs were bilaterally injected into a total of four sites at the VTA (AP: −3.0 mm, LM: ± 0.5 and ±0.2 mm, depth: 4.2 mm). An optical fiber (0.2 or 0.4 mm diameter) attached to a stainless steel ferrule (CFM14L10, Thorlabs) was implanted over the VTA using a cannula holder (XCF, Thorlabs). The optical fiber cannula was permanently cemented to the skull.

### Acute slice preparation

Mice were perfused under isoflurane anesthesia with ice-cold dissection buffer containing (in mM): 87 NaCl, 25 NaHCO_3_, 25 D-glucose, 2.5 KCl, 1.25 NaH_2_PO_4_, 0.5 CaCl_2_, 7 MgCl_2_, and 75 sucrose, aerated with 95% O_2_ + 5% CO_2_. The mice were then decapitated, and the brain was isolated and cut into 200-μm-thick horizontal sections on a vibratome in the ice-cold dissection buffer. The slices containing the VTA were incubated for 30 min at 35 °C in the dissection buffer and maintained thereafter at RT in standard artificial cerebrospinal fluid (aCSF) containing (in mM): 125 NaCl, 25 NaHCO_3_, 25 D-glucose, 2.5 KCl, 1.25 NaH_2_PO_4_, 1 MgCl_2_, and 2 CaCl_2_), aerated with 95% O_2_ and 5% CO_2_.

### In vitro electrophysiology

Whole-cell patch-clamp recordings from cultured HEK 293 cells or neurons in acute brain slices were performed using an IPA amplifier (Sutter Instruments) at RT. Fluorescently labeled cells were visually identified using an upright microscope (BX51WI; Olympus) equipped with a scientific complementary metal-oxide-semiconductor (sCMOS) video camera (Zyla4.2plus; Andor). The recording pipettes (5 − 7 MΩ) were filled with the intracellular solution containing (in mM): 135 potassium gluconate, 4 KCl, 4 Mg-ATP, 10 Na_2_-phosphocreatine, 0.3 Na-GTP, and 10 HEPES (pH 7.3, 280 mOsmol/l). Patch pipettes (5–7 MΩ) had a series resistance of 6.5–25 MΩ, which was compensated to have a final value of 6.5–7.0 MΩ for voltage-clamp recordings^51^. Data were filtered at 5 kHz, digitized at 10 kHz, and recorded using the SutterPatch software running on Igor Pro 8. For measuring photocurrents of depolarizing opsins, cells were voltage-clamped at −60 mV (HEK 293 cells and DA neurons) or −70 mV (MS neurons). For measuring the photocurrents of GtACR1 and GtACR2, cells were voltage-clamped at 0 mV (HEK 293 cells) or −30 mV (DA neurons). For measuring the photocurrents of ArchT or eNpHR3.0, cells were voltage-clamped at −20–0 mV to achieve near-zero holding currents. For current-clamp recordings from DA neurons, the membrane potentials were held at around −60 mV to prevent spontaneous firing unless otherwise noted. For testing the inhibitory effect of stGtACR1 activation (Fig. 2h, i), spikes at 5–10 Hz were evoked by current injections. In some experiments (Fig. 2e, f, j and k, Supplementary Fig. 5, and Supplementary Fig. 6e, f), neurons were current-clamped at around −40 mV to induce spontaneous APs. In such a case, some neurons were highly adaptive to the depolarized membrane potentials and showed little spontaneous firings. We, therefore, chose the cells that robustly exhibited a spontaneous AP rate of more than 1 Hz for further analyses. The PL intensity of the specimen was measured using a photodiode sensor that was routinely calibrated for each experiment.

### Conditioned place preference test

More than 2 weeks after AAV injection, mice were bilaterally injected with SMPs (50 mg/ml in Ringer’s solution) and used for behavioral tests at >1 week after SMP injection. Conditioned place preference (CPP) tests were performed in an X-ray machine (MX-160Labo, mediXtec Japan) under white LED lights at the dark period of the 12/12-h light/dark cycle. We used two types of test chambers. Both chambers had two compartments with different floor textures, only one of which was irradiated with X-rays. To restrict the X-ray irradiation (X-irradiation) to one side, the other compartment was shielded with lead boards (except for a small gate between the two compartments). Because phasic stimulation to VTA-DA neurons is known to be effective to induce place preference^27^, we designed one of the chambers to introduce pulsed X-irradiation through an X-ray chopper wheel in one component (Fig. 5b: Chamber I). We designed another chamber to introduce continuous X-irradiation in one compartment (Fig. 5d) because tonic inhibition of VTA-DA neurons effectively induces place aversion^29^. The test chamber for continuous X-irradiation had different visual cues on the wall of each compartment in addition to different floor textures (Fig. 5d: Chamber II).

For CPP tests using ChRmine-expressing mice and corresponding control mice, mice were first habituated to the Chamber I (15 min/day, three sessions). On the first day of the tests, mice were placed in the test chamber and allowed to freely explore for 10 min without X-irradiation. On the following 4 days, the mice were locked in either of the two compartments for 15 min each and received pulsed X-irradiation only in one compartment (150 kV, 3 mA; 50 ms duration, 10 pulses at 10 Hz, every 30 s), which we call “Pulsed conditioning” or PC (Fig. 5c). On the sixth day, the mice were placed in Chamber I and freely explore for 10 min without X-irradiation. For CPP tests using stGtACR1-expressing mice and corresponding control mice, the mice were habituated to the Chamber II for 5 min and then allowed to explore freely for 10 min without X-irradiation on the first day. On the second and third days, the mice were conditioned for 10 min with freely moving in the chamber where only one of the compartments received continuous X-irradiation (150 kV, 3 mA), which we call “Free moving conditioning” or FC (Fig. 5e). On the fourth day, the mice were placed in Chamber II for 10 min without X-irradiation. On the first and last day of the tests, the mice were videotaped at an oblique angle using a USB camera while the whole chamber was illuminated by ambient white LED light. Movies taken by the camera were displayed on a computer screen using Amcap and recorded online using Wondershare Filmora scrn. The ears of mice were tracked offline using DeepLabCut^52^. After completion of the CPP tests, the mice have perfused with 4% PFA and the brain was post-fixed overnight. The brain was cut into coronal sections (section thickness: 80 μm) and the SMP injection sites were observed. The traces of the SMP injections were found at ±0–200 μm away from the dorsal edge of VTA. In some cases, these injected SMPs were found along the injection track as well (Fig. 5a). Two mice that showed a biased preference for one chamber (>85%) on the first day were excluded from the analysis.

### Immunostaining

For immunostaining of brain slices, we performed transcardial perfusion and post-fixation overnight using 4% paraformaldehyde (PFA). The fixed brains were sectioned into coronal slices on a vibratome (section thickness: 80 μm) or using a cryostat (after immersion of the fixed brain in 30% sucrose solution for >2 days at 4 °C; section thickness: 40 μm). The slices were washed three times with a blocking buffer containing 1% bovine serum albumin (BSA) and 0.25% Triton-X in phosphate buffer saline (PBS) and then incubated with primary antibodies (anti-tyrosine hydroxylase, rabbit polyclonal, 1:1000, Merck Millipore; anti-Iba1, rabbit monoclonal, 1:500, Wako; anti-GFAP, mouse monoclonal, 1:1000, Merck Millipore; anti-NeuN, mouse monoclonal, 1:500, Merck Millipore; anti-mouse serum albumin, goat polyclonal, 1:1000, Abcam; anti-doublecortin, rabbit polyclonal, 1:1000, Abcam; anti-c-Fos, rabbit monoclonal, 1:1000, Abcam) in the blocking buffer overnight at 4 °C. Only for immunostaining of NeuN, the slices were incubated with the primary antibody for ~35–40 h at 4 °C. The slices were then washed three times with the blocking buffer and then incubated with secondary antibodies (CF594-conjugated or CF488A-conjugated donkey anti-rabbit IgG, 1:1000, Biotium; CF488A-conjugated goat anti-mouse IgG, 1:1000, Biotium; CF488A-conjugated donkey anti-goat IgG, 1:1000, Biotium) in the blocking buffer for 1–2 h at RT. Cellular nuclei were stained by incubation for 10–15 min with DAPI (2 μM in phosphate buffer) or Hoechst 33342 (5 μg/ml in PBS) at RT. The stained samples were mounted using DABCO and observed under a fluorescence microscope (BZ-9000, Keyence) or confocal microscope (LSM710, Zeiss). Images were saved using BZ-X Viewer (Keyence) for epifluorescence imaging or using ZEN (Zeiss) for confocal imaging.

### In vivo X-irradiation

For cFos induction experiments, more than 2 weeks after AAV injection in the VTA of DAT-IRES-Cre mice, SMPs (50 mg/ml in Ringer’s solution) were injected at the same location as AAV injection. More than 1 week after SMP injection, mice were anesthetized with a combination anesthetic (0.3 mg/kg medetomidine hydrochloride, 4 mg/kg midazolam, 5 mg/kg butorphanol tartrate) and placed on a heating pad in the X-ray machine. The head of the mice was targeted for X-irradiation at the dose rate of 0.5 or 1 Gy/min (150 kV, 3 mA; 1 min pulses, every 2 min, 5 times). Mice were perfused with 4% PFA at 2 h after X-irradiation.

For assessment of radiation toxicity, mice were irradiated with X-rays as described below: (1) Pulsed X-ray radiation: mice were placed in the X-irradiated compartment of Chamber I and received pulsed X-irradiation (150 kV, 3 mA, ~0.5 Gy/min; 50 ms, 10 Hz, 10 pulses/train, every 30 s; 30 trains/day, 4 days), corresponding to the “Pulsed conditioning” (PC). For 2×, 3×, or 4× PC radiation (Fig. 6), the mice were irradiated with pulsed X-ray radiation with 20, 30, or 40 pulses, respectively, for each radiation train; (2) Fractionated X-ray radiation: mice were placed in the X-irradiated compartment of Chamber II and received fractionated X-irradiation corresponding to the “Free moving conditioning” (150 kV, 3 mA, ~0.7 Gy/min; 1 min pulses, every 2 min; 5 pulses/day, 2 days); and (3) Acute high-dose radiation: mice were placed in a small box to receive a high dose X-irradiation (150 kV, 3 mA, 1.35 Gy/min; 400 s pulse, once). Control mice did not receive any radiation.

### EdU staining

To identify newly generated cells in the hippocampus, 5-ethynyl-2′-deoxyuridine (EdU) staining was performed using Click-iT EdU Alexa Fluor 488 Imaging Kit (Thermo Fisher Scientific) following the manufacturer’s protocol. EdU dissolved in Ringer’s solution (10 mg/ml) was intraperitoneally administered for 6 consecutive days starting at 28 days after the last fraction of X-irradiation. The mice were transcardially perfused with 4% PFA at 28 days after the first day of EdU injection. The brain was post-fixed overnight and then immersed in 30% sucrose solution for >2 days at 4 °C. Coronal sections (thickness: 40 μm) were cut using a cryostat. The sections were incubated with 0.5% TritonX-100 in PBS for 30 min at RT and washed 2 times with PBS containing 3% BSA. The sections were then incubated with Click-iT reaction cocktail for 60 min at RT and washed 3 times with PBS containing 3% BSA. The sections were subsequently subjected to immunostaining of Iba-1 and NeuN with the protocol described above.

### TUNEL assay

For detection of apoptotic cells, TdT-mediated dUTP Nick-End Labeling (TUNEL) assay was performed using Click-iT Plus TUNEL Assay with Alexa Fluor 488 (Thermo Fisher Scientific) following the manufacturer’s protocol. Briefly, mice have transcardially perfused with 4% PFA and the brain was post-fixed overnight. The brain was then immersed in 30% sucrose solution for >2 days at 4 °C. The brain was cut into coronal sections using a cryostat (section thickness: 10 μm). The sections were permeabilized with proteinase K for 20 min at 37 °C and then incubated with the TdT reaction buffer for 20 min at 37 °C. The sections were then incubated with the TdT reaction mixtures for 120 min at 37 °C, followed by blocking with 3% BSA and 0.5% Triton X-100 in PBS for 20 min at RT. The section was then incubated with the Click-iT Plus TUNEL reaction cocktail for 60 min at 37 °C. For DNA staining, the sections were incubated with a Hoechst 33342 solution (5 μg/ml in PBS) for 15 min at RT. After washing, the tissue sections were mounted using DABCO.

### FACS analysis of bone marrow cells

Two long bones (femur and tibia) were isolated per mouse and bone marrow cells were flushed out with 10 ml of FACS buffer (2% FBS, 2 mM EDTA in PBS) using a syringe with a 25 G needle. Cells were treated with 1 ml of ACK buffer (150 mM NH_4_Cl, 10 mM KHCO_3_, 0.1 mM Na_2_EDTA in water) for 1 min, centrifuged at 470×*g* for 3 min and resuspended in 2 ml of FACS buffer. Live cells were counted with 0.4% (w/v) Trypan Blue Solution (FUJIFILM Wako Chemicals) and were stained at 20 μl of FACS buffer per 1 × 10^6^ cells containing the first set of following antibodies (all from Biolegend, unless specified otherwise): biotinylated hematopoietic lineage antibodies against NK1.1 (clone PK136), CD11b (M1/70), Ter119 (Ter119), Gr-1 (RB6-8C5), CD4 (GK1.5), CD8α (53-6.7), CD3ε (145-2C11), B220 (RA3-6B2), and IL-7Rα (SB/199) (all 1:500 dilution); BV510-conjugated anti-CD16/32 (93, 1:50), PE-Cy5-conjugated anti-CD135 (A2F10, 1:100), and AF700-conjugated anti-CD48 (HM48-1, 1:100) in FACS buffer for 30 min on ice. Cells were washed with FACS buffer once and stained with the second set of following antibodies: Pacific blue-conjugated streptavidin (1:100, Thermo Fisher Scientific), AF488-conjugated anti-CD150 (TC15-12F12.2, 1:100), PE-conjugated anti-EPCR (eBio1560, 1:100, Thermo Fisher Scientific), PE-Cy7-conjugated anti-c-Kit (2B8, 1:100), APC-conjugated anti-CD34 (HM34, 1:50) in FACS buffer for 90 min on ice. Tubes were tapped every 30 min to ensure dispersion of antibodies. Cells were washed twice and resuspended in FACS buffer containing Hoechst (2 μg/ml, Thermo Fisher Scientific) and filtered with a 77 μm nylon filter prior to acquisition with the flow cytometer FACSAria^TM^ III (Becton, Dickson, and Company). Data were analyzed using the FlowJo software (Becton, Dickson, and Company).

### Data analysis

The number of HEK 293 cells in 35-mm culture dishes (Supplementary Fig. 9) was counted by randomly selecting 100 μm × 100 μm squares surrounding the Ce:GAGG crystal for the dishes containing the crystal or anywhere on the coverslip for control dishes. The mean cell density was calculated as the average of the cell densities of three sites per dish.

To estimate the survival rate of dissociated hippocampal neurons (Fig. 4a), we randomly selected three 0.2 mm × 1.0 mm rectangles per dish, surrounding the Ce:GAGG crystal (one of the longer sides of the rectangle was attached to the edge of the crystal) for the dishes containing the crystal or anywhere on the coverslip for control dishes. The dissociated neurons within these sites at DIV1 were monitored at DIV2, 4, and 7.

To quantify the glial accumulation in epi-fluorescence images (Supplementary Fig. 10a, b), binary images were obtained by thresholding. A 100 μm × 100 μm square was drawn around the trace of the SMPs or the optical fiber (one of the sides of the square was attached to the edge of the trace; three sites per image), and the number of pixels exceeding the threshold in these squares was calculated using ImageJ. Likewise, the average number of NeuN-positive cells (Fig. 4b–d) in 100 μm × 100 μm squares drawn around the SMP/optical fiber trace in confocal images (three sites per image) was calculated using ImageJ.

To analyze animal movements in the CPP tests using a Python package of DeepLabCut^52^, the left and right ears of mice were manually annotated using 20 − 80 frames per movie to train a deep neural network. The animal trajectories and heat maps (Fig. 5g, i) were generated using custom-made Matlab programs. An estimated ear position with a low likelihood (<0.8) was omitted and replaced with a pixel value obtained using linear interpolation of neighboring values. The middle pixel coordinate between the tracked left and right ears were considered the animal trajectory, assuming that the coordinate represented the location of the mouse head. The heat maps shown in Fig. 5g, i indicate the probability of the presence of the animal trajectory within each 100 × 100-pixel images. In the CPP tests, two mice that showed a substantially biased preference for one of the two compartments (>85%) on the first day were excluded from the analysis.

All values are expressed as mean ± SEM. Statistical tests were performed using GraphPad Prism or Igor Pro. The normality of data distribution was routinely tested. Analyses of two-sample comparisons were performed using unpaired or paired *t*-tests when each sample was normally distributed, or Mann–Whitney U tests when at least one of the samples in every two-sample comparison was not normally distributed. Tests for two-sample comparison were two-sided. Statistical analyses for multiple comparisons were carried out using one-way or two-way ANOVA followed by Bonferroni’s multiple comparison tests or Dunnett’s multiple comparison tests vs. the control unless otherwise noted.

### Reporting summary

Further information on research design is available in the Nature Research Reporting Summary linked to this article.

## Supplementary information

Supplementary Information

Peer Review File

Reporting Summary

## Data Availability

The data used to generate figures that support the findings of this study are freely available in the Open Access CERN database Zenodo: https://zenodo.org/communities/ty-lab-data with DOI hyperlink: 10.5281/zenodo.4964867. Source data are provided with this paper.

## Source data

Source Data

## Code availability

The Matlab codes used to generate Figs. 3b and 5f–i are freely available on GitHub: https://github.com/juniti-y/Matsubara_et_al_Nat_Commun_2021.
