# Author Correction: Remote control of neural function by X-ray-induced scintillation

**DOI:** 10.1038/s41467-022-29664-z

**Published:** 2022-04-06

**Authors:** Takanori Matsubara, Takayuki Yanagida, Noriaki Kawaguchi, Takashi Nakano, Junichiro Yoshimoto, Maiko Sezaki, Hitoshi Takizawa, Satoshi P. Tsunoda, Shin-ichiro Horigane, Shuhei Ueda, Sayaka Takemoto-Kimura, Hideki Kandori, Akihiro Yamanaka, Takayuki Yamashita

**Affiliations:** 1 Department of Neuroscience II, Research Institute of Environmental Medicine, Nagoya University, Nagoya, Japan; 2 Department of Neural Regulation, Graduate School of Medicine, Nagoya University, Nagoya, Japan; 3 Department of Physiology, Fujita Health University School of Medicine, Toyoake, Japan; 4 Nara Institute of Science and Technology, Nara, Japan; 5 Department of Computational Biology, Fujita Health University School of Medicine, Toyoake, Japan; 6 International Research Center for Medical Sciences, Kumamoto University, Kumamoto, Japan; 7 Department of Life Science and Applied Chemistry, Nagoya Institute of Technology, Nagoya, Japan; 8 PRESTO, Japan Science and Technology Agency, Kawaguchi, Japan; 9 Department of Neuroscience I, Research Institute of Environmental Medicine, Nagoya University, Nagoya, Japan; 10 Department of Molecular/Cellular Neuroscience, Graduate School of Medicine, Nagoya University, Nagoya, Japan; 11 CREST, Japan Science and Technology Agency, Kawaguchi, Japan

**Keywords:** Behavioural methods, Optogenetics, Fear conditioning

Correction to: *Nature Communications* 10.1038/s41467-021-24717-1, published online 22 July 2021.

The original version of this Article contained an error in Figure 3d. The label ‘ChRmine-eYFP’ was incorrectly shown in orange font instead of green font. This error has been corrected in the HTML and PDF versions of the Article.

