# Supplementary material for: Remote control of neural function by X-ray-induced scintillation
Source: Nat Commun. 2021 Jul 22;12:4478. doi: 10.1038/s41467-021-24717-1 (PMC8298491; doi:10.1038/s41467-021-24717-1)
Supplement: Supplementary file 1 — Supplementary Information [file 41467_2021_24717_MOESM1_ESM.pdf]

Supplementary Information

Remote control of neural function by X-ray-induced scintillation

Takanori Matsubara, Takayuki Yanagida, Noriaki Kawaguchi, Takashi Nakano, Junichiro Yoshimoto, Maiko Sezaki, Hitoshi Takizawa, Satoshi P. Tsunoda, Shin-ichiro Horigane, Shuhei Ueda, Sayaka Takemoto-Kimura, Hideki Kandori, Akihiro Yamanaka, and Takayuki Yamashita.

Correspondence to: takayuki.yamashita@fujita-hu.ac.jp (T.Yamashita)

Contents of the Supplementary Information:

Supplementary Figure 1 – Tissue penetration by X-rays, NIR, and blue light

Supplementary Figure 2 – Thermal effects of X-rays, NIR, and blue light

Supplementary Figure 3 – Activation of BeGC1 by Ce:GAGG PL

Supplementary Figure 4 – Cell-type-specific expression of ChRmine and stGtACR1

Supplementary Figure 5 – Activation of VTA-DA neurons by low-intensity PL

Supplementary Figure 6 – Activation of medial septum neurons by Ce:GAGG PL

Supplementary Figure 7 – Intensity of Ce:GAGG RL

Supplementary Figure 8 – Immunoreactivity against c-Fos in control mice

Supplementary Figure 9 – Proliferation of HEK293 cells around a Ce:GAGG crystal

Supplementary Figure 10 – Further analysis of Ce:GAGG biocompatibility

Supplementary Figure 11 – X-ray dose rates in the CPP test chambers

Supplementary Figure 12 – Locomotor behavior after whole-body X-irradiation

Supplementary Figure 13 – Test for BBB leakage in the X-irradiated brains

Supplementary Figure 14 – Body weight and hippocampal neurogenesis after X-irradiation

Supplementary Figure 15 – TUNEL analysis of the hippocampus after X-irradiation

Supplementary Figure 16 – FACS analysis of the bone marrow irradiated with X-rays

Supplementary Table 1– PCR primers used in this study

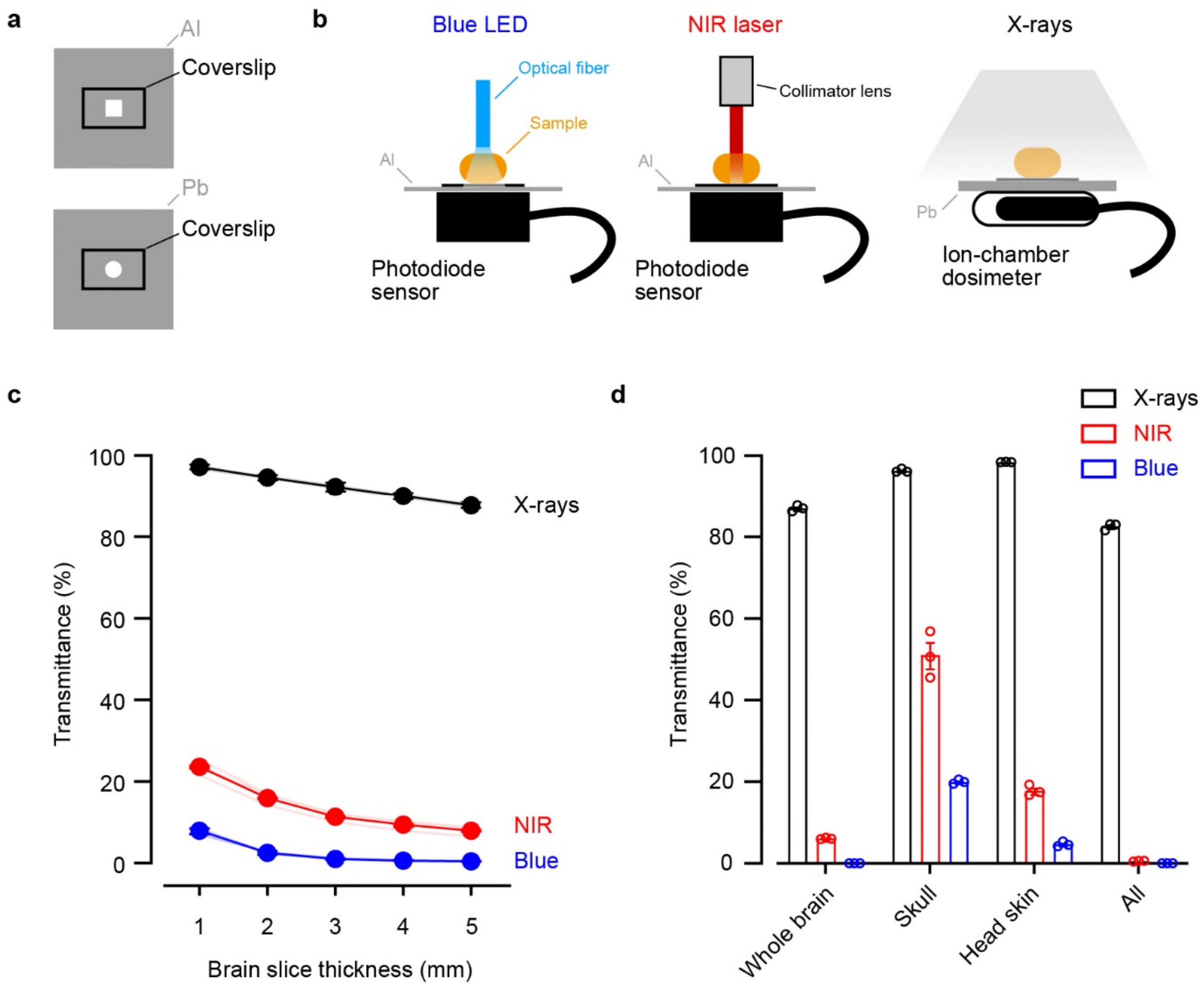

Supplementary Fig. 1 Tissue penetration by X-rays, NIR, and blue light. a The tissue penetration efficacy of different electromagnetic waves was measured using an aluminum (Al, top) or lead (Pb, bottom) plate with a hole shaped as a square (5 mm × 5 mm, top) or a circle (5 mm diameter, bottom) with a coverslip. The lead board was 3 mm thick. **b** Schematic of the experiments. The photosensor or dosimeter was placed under the hole in the plate. **c** Transmittance of brain slices of different thickness irradiated with X-rays (black, 150 kV, 3 mA; input: 0.116−0.122 Gy/min), NIR laser (red, 976 nm; input: 42.8−43.9 mW/cm^2) and blue LED (blue, 470 nm; input: 48.6−49.5 mW/cm^2) (n = 3 slices for each group). **d** Transmittance of the shaved head skin, skull, and whole brain of mice (n = 3 mice for each group). Samples were placed horizontally. Input power: 0.129−0.131 Gy/min for X-rays; 43.1−45.1 mW/cm^2 for NIR; 51.3 mW/cm^2 for blue LED. Open circles and lightly colored lines indicate individual data. Values are mean ± SEM. Source data are provided as a Source Data file.

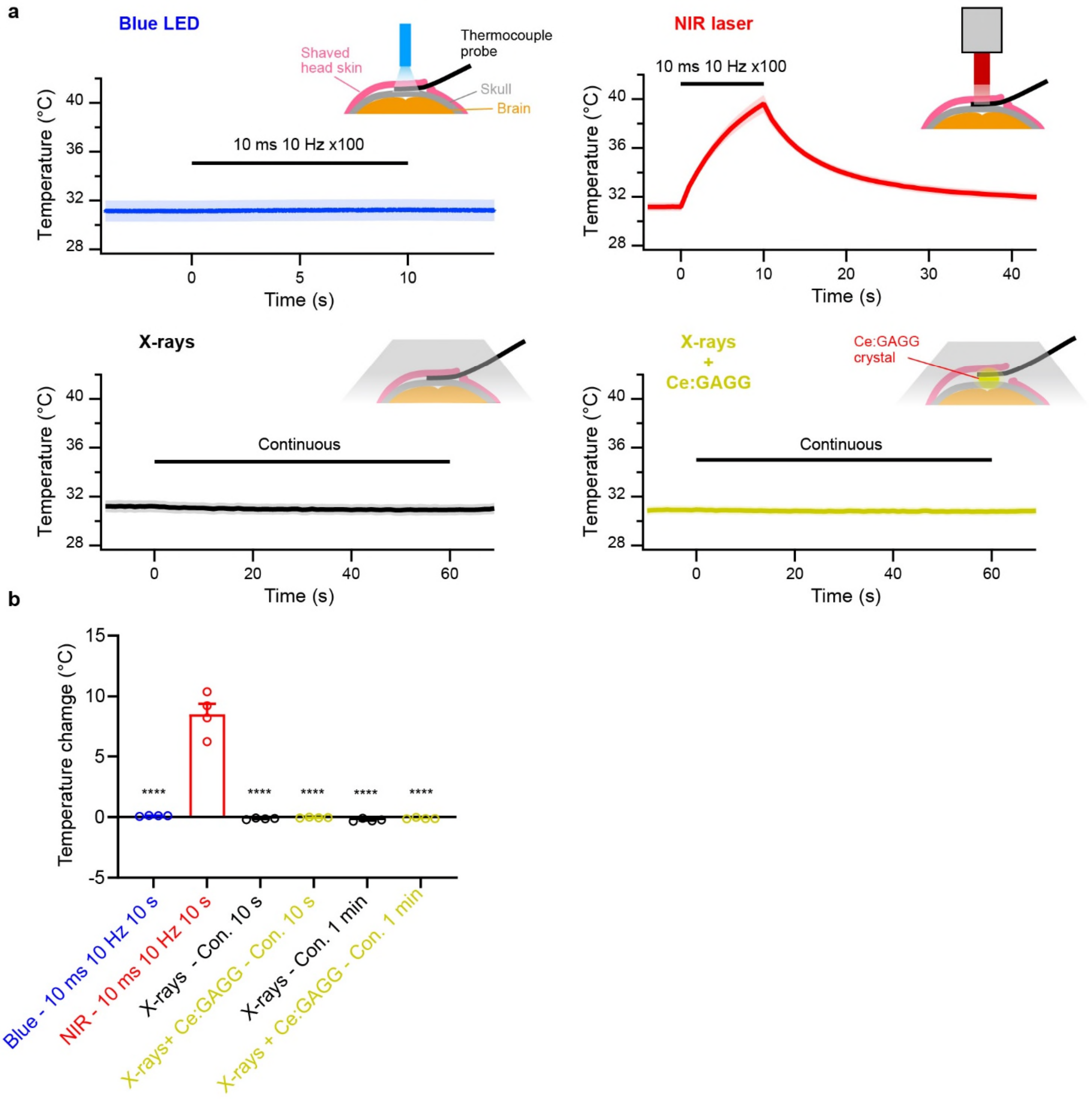

Supplementary Fig. 2 Temperature under the skin of the head irradiated by X-rays, NIR, or blue light. a Average traces of temperature recordings (n = 4 mice each). The temperature between the shaved head skin and skull of anesthetized mice irradiated with blue LED (10 mW at the fiber tip; fiber diameter, 400 μm), NIR laser (200 mW/mm^2 after a collimator lens with a beam diameter of 4 mm) or X-rays (150 kV, 3 mA, 1.35 Gy/min) was measured. Scintillation-induced temperature changes were measured by placing a Ce:GAGG crystal (6 mm × 4 mm × 1 mm) together with the temperature sensor between the skin of the head and the skull. The anesthetized mouse was placed on a heat pad. Thick line: mean, Shadows: ± SEM. **b** Temperature changes under various conditions (n = 4 mice each). Con.: continuous. ****p < 0.0001 for all pairs; Dunnett's multiple comparison test, vs. NIR, two-sided. Open circles indicate individual data. Values are mean ± SEM. Source data are provided as a Source Data file.

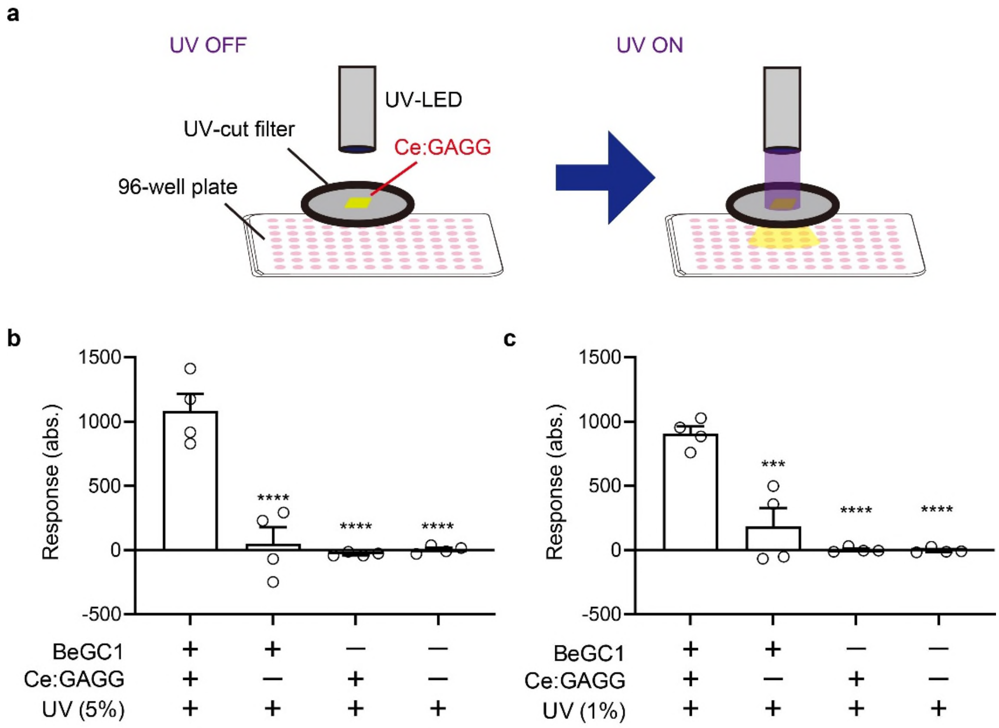

Supplementary Fig. 3 Ce:GAGG photo-luminescence activates the enzyme rhodopsin BeGC1. **a** Experimental setup. A Ce:GAGG crystal (6 mm × 4 mm × 1 mm) was irradiated by UV (1% or 5% intensity of the maximum LED power) to illuminate HEK 293 cells expressing BeGC1 or control cells cultured in 96-well plates. PL intensity: 1.56 μW/mm^2 for 5% UV; 0.66 μW/mm^2 for 1% UV. UV irradiation was largely attenuated by a UV-cut filter placed over the plate. The same wells were irradiated with UV only through the UV-cut filter. **b** BeGC1 activation was quantified by measuring the luminescence intensity derived from Glosensor (n = 4 wells each). Response amplitude was calculated by substituting the intensity at 8−12 min after 5% UV irradiation from the baseline. abs., absolute value of luminescence intensity. ****p < 0.0001 for all pairs; Bonferroni's multiple comparison test, vs. the BeGC1(+)/Ce:GAGG(+)/UV(+) group, two-sided. **c** Same as **b**, but with 1% UV irradiation (n = 4 wells each). BeGC1(+)/Ce:GAGG(–)/UV(+), ***p = 0.0002; BeGC1(–)/Ce:GAGG(+)/UV(+), ****p < 0.0001; BeGC1(–)/Ce:GAGG(–)/UV(+), ****p < 0.0001; Bonferroni's multiple comparison test, vs. the BeGC1(+)/Ce:GAGG(+)/UV(+) group, two-sided. Open circles indicate individual data. Values are mean ± SEM. Source data are provided as a Source Data file.

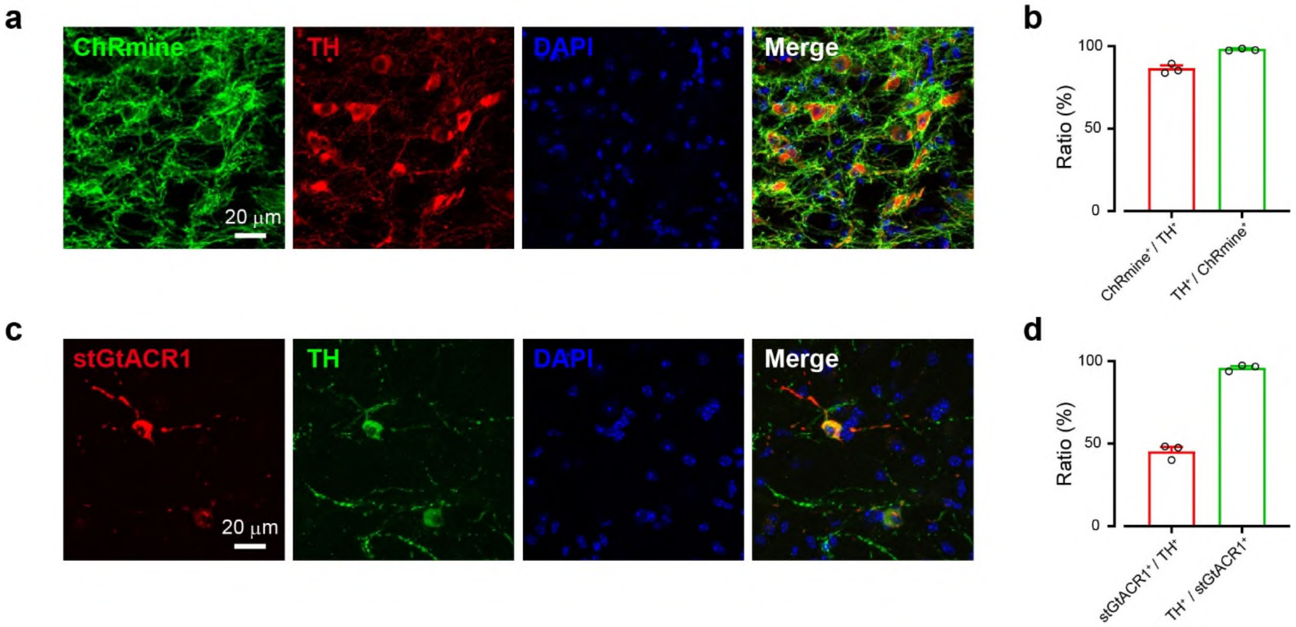

Supplementary Fig. 4 DA neuron-specific expression of ChRmine and stGtACR1. a Representative confocal images showing virally induced expression of ChRmine-eYFP (green) and immunostained TH (red) in the VTA. Blue:DAPI. **b** Quantification of overlapping of ChRmine-eYFP- and TH-labelled neurons in the VTA (*n* = 3 mice). **c** Same as **a**, but for stGtACR1-FusionRed (red) and immunostained TH (green). **d** Same as **b**, but for stGtACR1-FusionRed- and TH-labelled neurons (*n* = 3 mice). Open circles indicate individual data. Values are mean ± SEM. Source data are provided as a Source Data file.

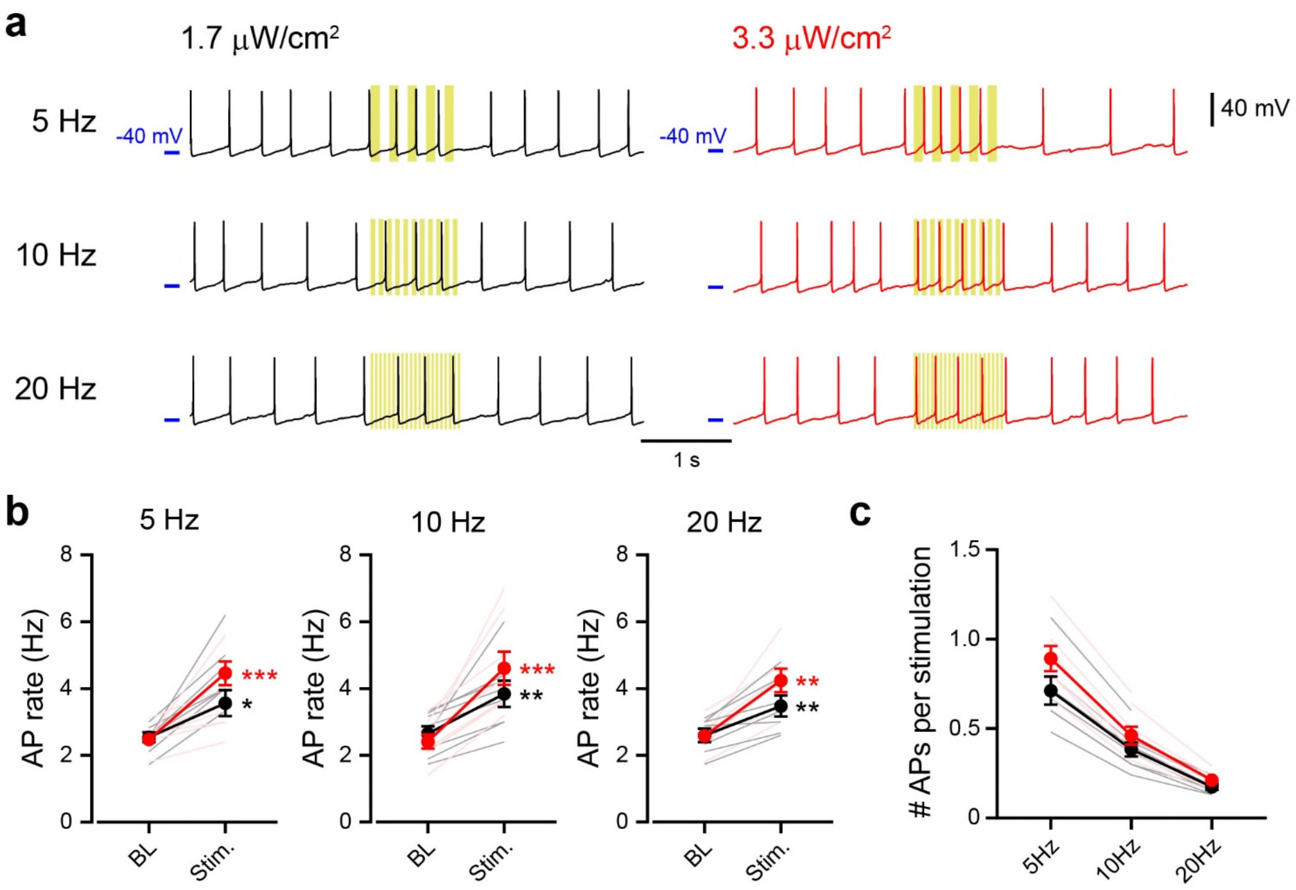

Supplementary Fig. 5 Activation of VTA-DA neurons by low-intensity PL. a Example recordings from a ChRmine-expressing VTA-DA neuron illuminated with pulses of Ce:GAGG PL (yellow bars) at 1.7 (left) and 3.3 (right) μW/cm^2. 5 Hz: 100 ms, 5 pulses at 5 Hz; 10 Hz: 50 ms, 10 pulses at 10 Hz; 20 Hz: 25 ms 20 pulses at 20 Hz. **b** Quantification of AP rates at the baseline (BL) and during pulsed PL illumination (Stim) at 1.7 (black) and 3.3 (red) μW/cm^2 (5 Hz, n = 7 cells for each; 10 Hz, n = 8 cells for each; 20 Hz, n = 7 cells for each). 5 Hz, 1.7 μW/cm^2, *p = 0.0268; 5 Hz, 3.3 μW/cm^2, ***p = 0.0007; 10 Hz, 1.7 μW/cm^2, **p = 0.0064; 10 Hz, 3.3 μW/cm^2, ***p = 0.0008; 20 Hz, 1.7 μW/cm^2, **p = 0.0051; 20 Hz, 3.3 μW/cm^2, **p = 0.0023; paired t tests vs. BL, two-sided. **c** Quantification of the number of APs per stimulation at 1.7 (black) and 3.3 (red) μW/cm^2 at different frequencies (5 Hz, n =7 cells for each; 10 Hz, n = 8 cells for each; 20 Hz, n = 7 cells for each). Lightly colored lines indicate individual cells Values are mean ± SEM. Source data are provided as a Source Data file.

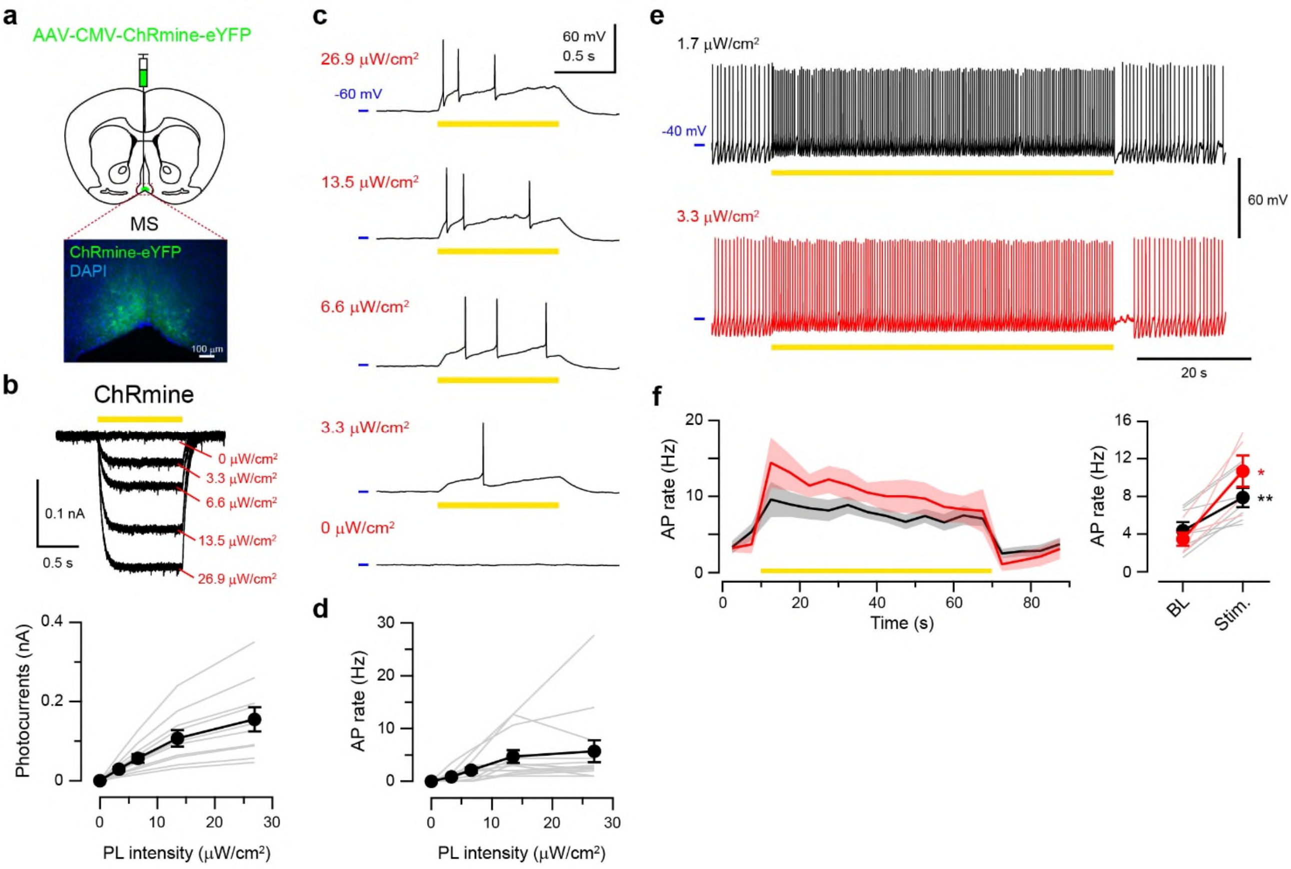

Supplementary Fig. 6 Activation of medial septum neurons by Ce:GAGG PL a Schematic of the experiment. Inset, an epifluorescent image of neurons expressing Chmine-eYFP (Green) in the ventral part of the medial septum (MS). Blue: DAPI. Similar results were obtained in 3 mice. **b** Top, sample voltage-clamp recordings from a ChRmine-expressing MS neuron responding to 1-s Ce:GAGG PL. Bottom, photocurrent amplitude vs. PL intensity (*n* = 10 cells). **c** Sample recordings from a ChRmine-expressing MS neuron current-clamped at approximately -60 mV, responding to 1-s PL. **d** AP rate of ChRmine-expressing neurons vs. PL intensity (*n* = 13 cells). Irradiation of PL at 3.3 (red) μW/cm^2 elicited APs in 9 out of 13 neurons. **e** Sample recordings from a ChRmine-expressing neuron current-clamped at approximately -40 mV, responding to 1-min PL illumination. **f** Time course (left) and quantification (right) of average AP rates with 1-min PL illumination at 1.7 (black) or 3.3 (red) μW/cm^2. Thick line: mean, Shadows: ± SEM, BL: baseline, Stim.: stimulation. Lightly colored lines indicate individual cells. 1.7 μW/cm^2: n = 7 cells, **p = 0.0017; 3.3 μW/cm^2: n = 5 cells, *p = 0.0143; paired t-test, two-sided. Values are mean ± SEM. Source data are provided as a Source Data file.

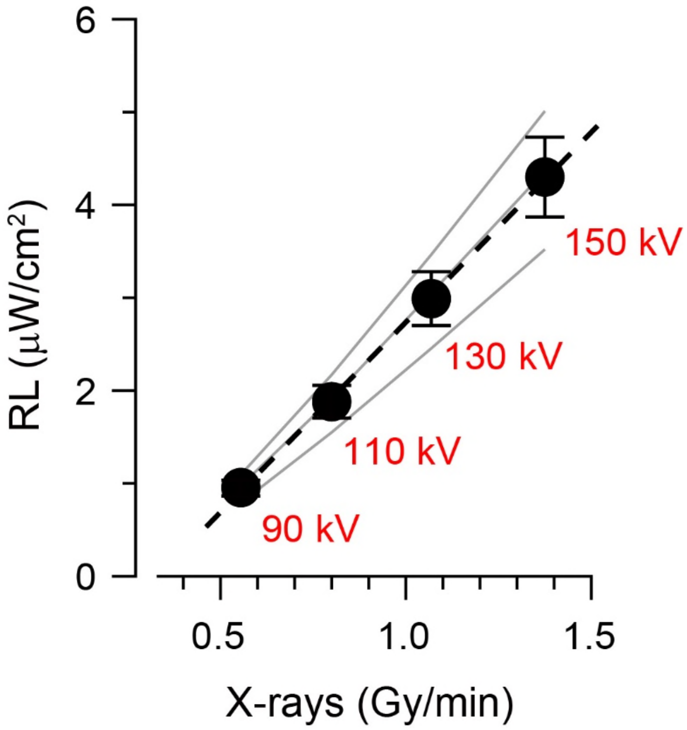

Supplementary Fig. 7 Intensity of Ce:GAGG RL. The intensities of RL emitted by SMPs were measured by fiber optics at different tube potentials of the X-ray source with a constant tube current (3 mA) (n = 3 samples). The X-ray dose rates at these tube potentials were measured by a dosimeter. These values in combination with the exponential decay of RL intensities in gray matter (measured as 32.75 % reduction by 200 μm distance) are used for simulation of a 3D map of RL emitted from a spherical aggregate of injected SMPs (Fig .3b). Gray lines indicate individual data. Values are mean ± SEM. Source data are provided as a Source Data file.

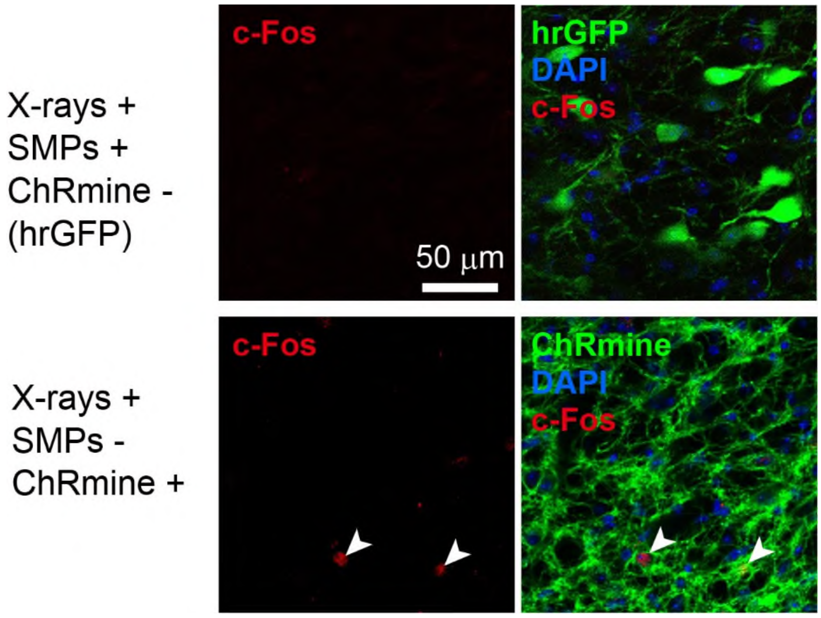

Supplementary Fig. 8 Immunoreactivity against c-Fos in control mice. Confocal images of immunoreactivity against c-Fos (red) of the VTA-DA neurons in control mice irradiated with X-rays (1.0 Gy/min, total 5 min). Representative images from a mouse with SMP injection but no ChRmine expression (top, hrGFP was expressed in the VTA-DA neurons) and that with ChRmine expression but no SMP injection (bottom) are shown. Similar results were obtained in 6 hemispheres from 3 mice for each group.

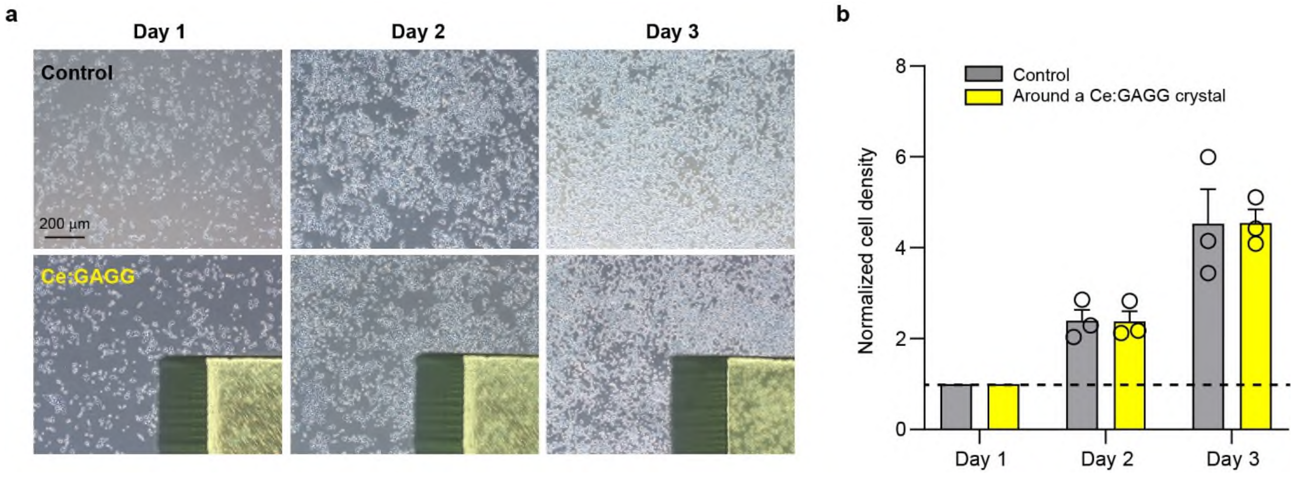

Supplementary Fig. 9 Proliferation of HEK293 cells around a Ce:GAGG crystal. a HEK293 cells were cultured in a dish with (bottom) or without (top) a Ce:GAGG crystal. **b** During 3 days of culturing, the proliferation rate of the cells at around the Ce:GAGG crystal (n = 3 dishes) did not differ significantly from that in the control dish (n = 3 dishes, $F_{1,4} = 0.000023$, $p = 0.996$; two-way ANOVA). Open circles indicate individual data. Values are mean ± SEM. Source data are provided as a Source Data file.

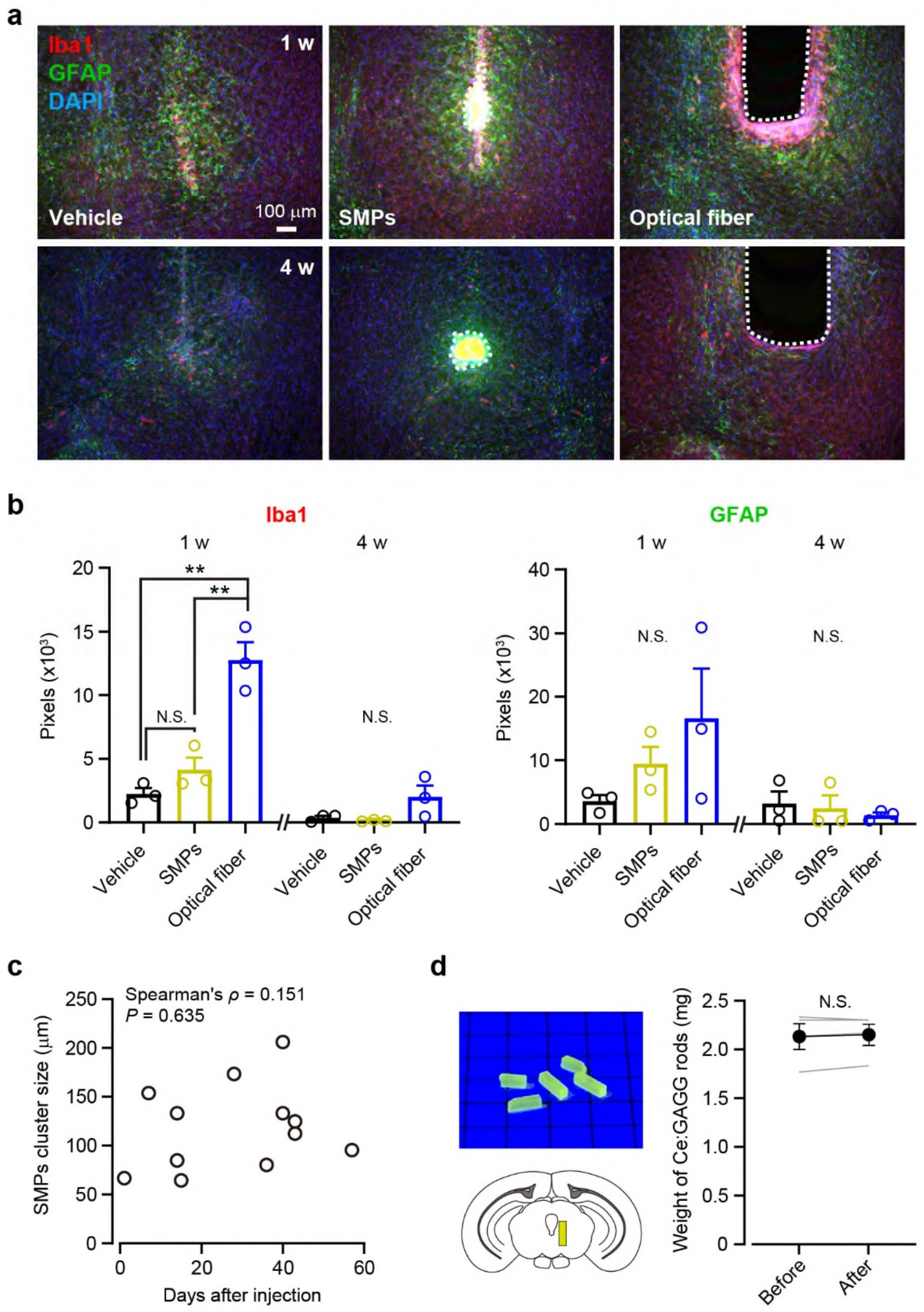

Supplementary Fig. 10 Further analysis of the biocompatibility of Ce:GAGG crystals. a Representative epi-fluorescence images of coronal slices showing immunostaining for activated microglia (Iba1, red) and astrocytes (GFAP, green) at the injection site of vehicle (left) or SMPs (middle), and at the ventral tip of an implanted optical fiber (right). Slices were obtained from the mice at one (1w, top) or four weeks (4w, bottom) after surgery. The trace of SMPs or an optical fiber (diameter, 400 µm) is outlined by a dashed line. Blue: DAPI. **b** Quantification of accumulation of microglial cells (Iba1, left) and astrocytes (GFAP, right) estimated in 100 µm x 100 µm squares near the injection/implantation traces (n = 3 mice for each group). Iba1-1w, $F_{2,6} = 28.93$, $p = 0.0008$; Iba1-4w, $F_{2,6} = 3.76$, $p = 0.0876$; GFAP-1w, $F_{2,6} = 1.85$, $p = 0.237$; GFAP-4w, $F_{2,6} = 0.326$, $p = 0.734$; one-way ANOVA. Iba1-1w: Vehicle vs. SMPs, $p = 0.734$; Vehicle vs. Optical fiber, $^{**}p = 0.0011$; SMPs vs. Optical fiber, $^{**}p = 0.0033$; Bonferroni's multiple comparison test, two-sided. **c** The injected SMP cluster sizes inspected at different days after injection (n = 12 mice, $\rho = 0.151$, $p = 0.635$; Spearman's

rank correlation, two-sided). **d** Left, an image of rod-shaped Ce:GAGG crystals under UV illumination (top) and schematic of the implantation (bottom). Grid scale, 2 mm. Right, the weight of the Ce:GAGG rods did not change after 4 w implantation (n = 4 mice, p = 0.495; paired t test, two-sided). N.S., not significant. Open circles indicate individual data. Values are mean ± SEM. Source data are provided as a Source Data file.

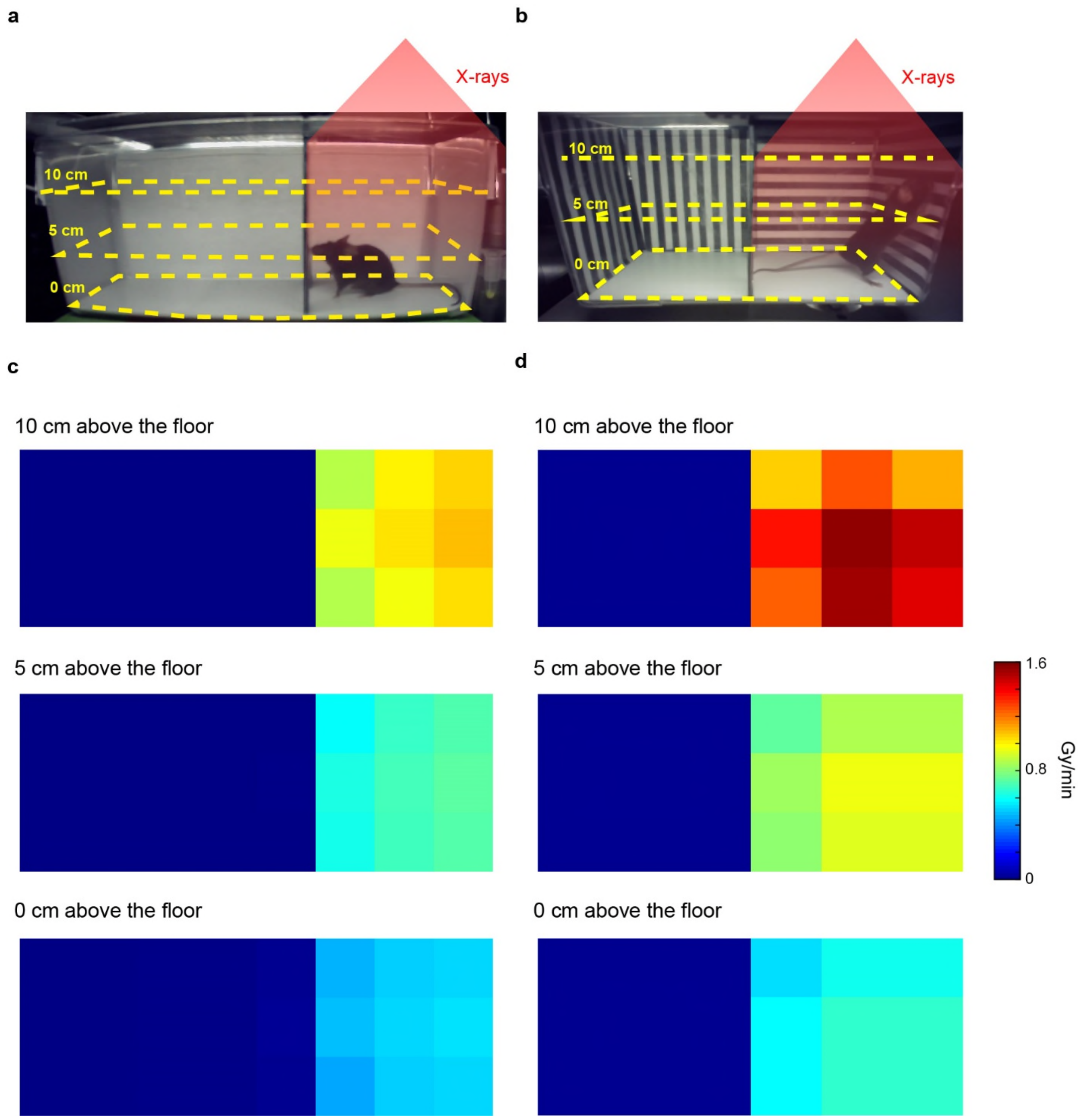

Supplementary Fig. 11 X-ray dose rates measured in the CPP test chambers. **a**, **b** X-ray dose rates were measured with a dosimeter placed at 0, 5 or 10 cm above the floor of Chamber I (**a**) and Chamber II (**b**). **c** Heat maps showing X-ray dose rates (in Gy/min) measured in both X-ray irradiated (right) and non-irradiated (left) compartments of Chamber I in the absence of the X-ray chopper. Section size: 3.3 cm × 3.2 cm. **d** Same as **c**, but for Chamber II. Section size: 3.3 cm × 4.0 cm**.** Source data are provided as a Source Data file.

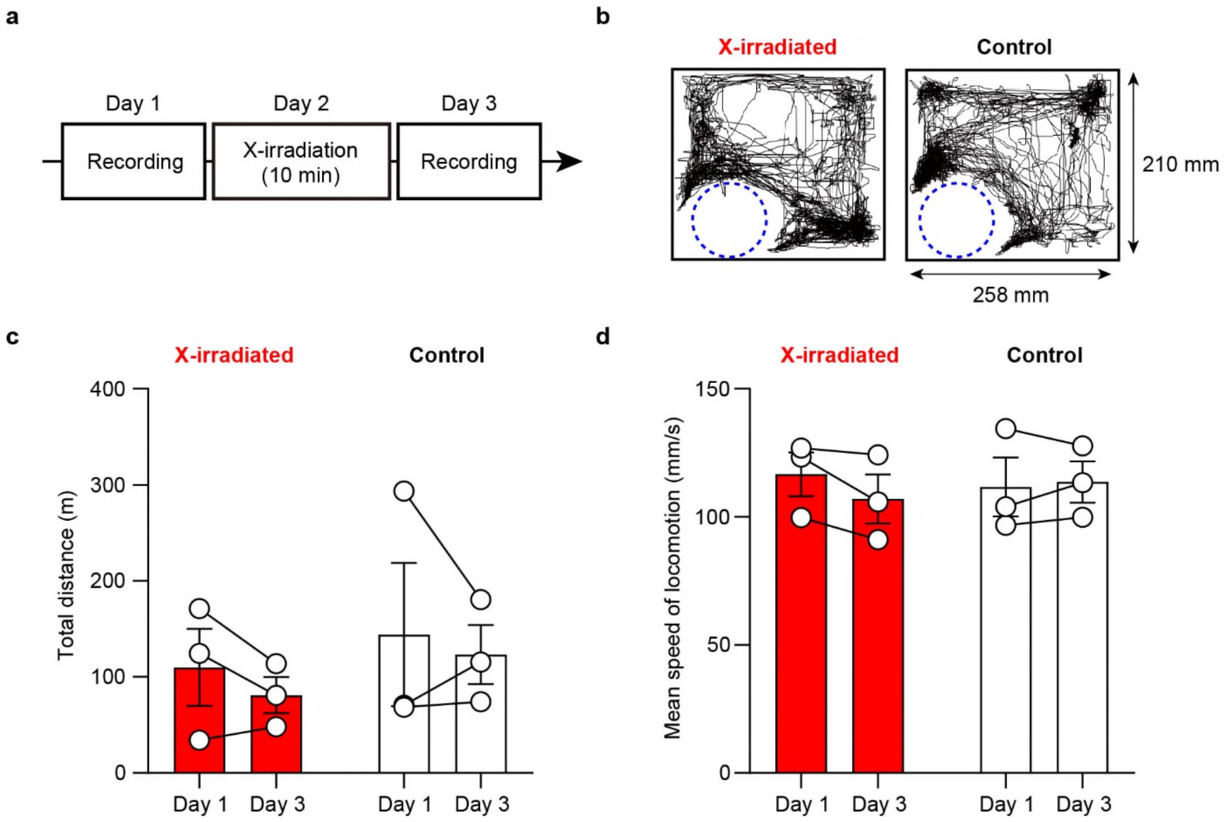

Supplementary Fig. 12 X-irradiation did not affect locomotor behavior at the home cage. a Schematic of the experiment. X-rays (150 kV, 3 mA) was irradiated onto anesthetized mice for 10 min in the X-irradiated compartment of the CPP test chamber for "Free moving" conditioning. **b** Top-view trajectory of X-irradiated (left) or non-irradiated (right, Control) mice during 1 h recording of their home-cage at day 3. One mouse per cage. The center of the mouse body was tracked using DeepLabCut[52]. The dotted circle indicates the location of a water bottle. **c**, **d** Total travel distance (**c**) and mean locomotion speed (**d**) of the mice at day 1 and day 3 (n = 3 mice each). The mean locomotion speed was calculated as the average values in the film frames where the tracked body location moved at >50 mm/s. **c**: X-irradiated, $p = 0.317$; Control, $p = 0.703$; paired t test, two-sided. **d**: X-irradiated, $p = 0.155$; Control, $p = 0.716$; paired t test, two-sided. Open circles indicate individual data. Values are mean ± SEM. Source data are provided as a Source Data file.

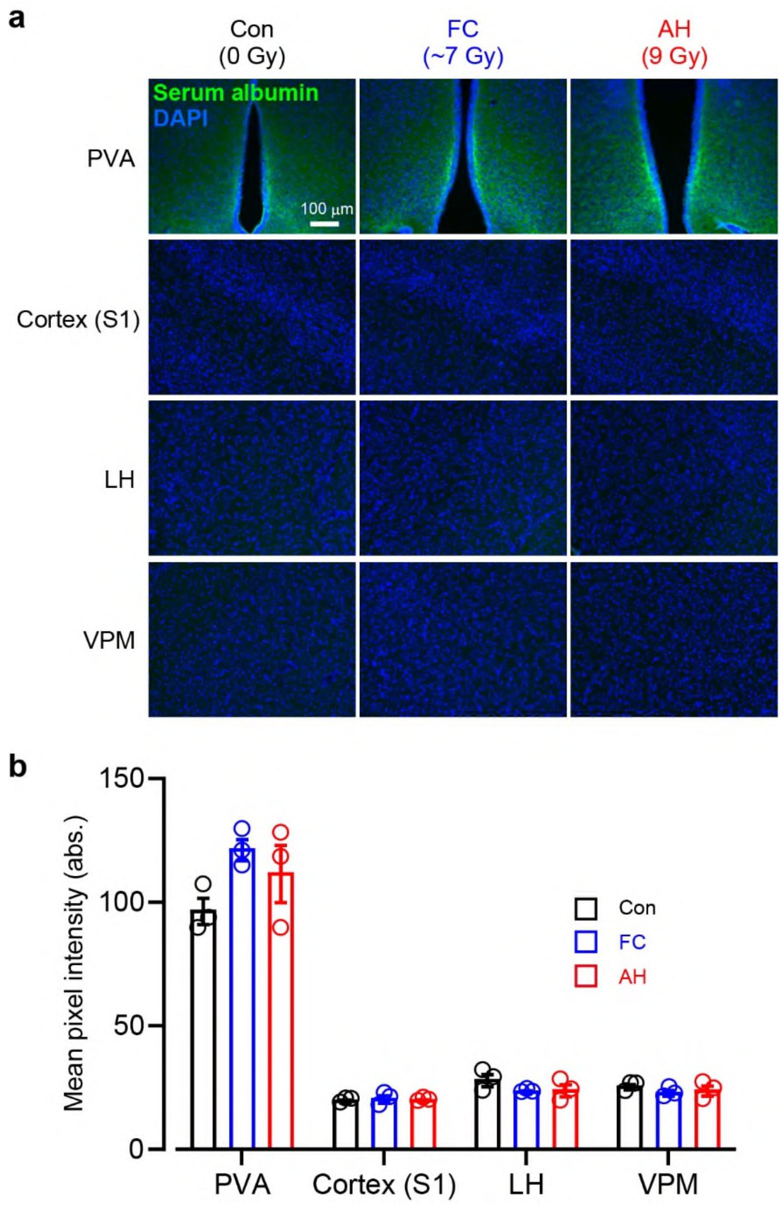

Supplementary Fig. 13 Immunoreactivity against serum albumin after X-irradiation. a To assess the brain blood barrier function, immunoreactivity against extravasated serum albumin was examined in the brain of mice perfused at 3 days after the last fraction of X-irradiation. Representative epi-fluorescence images show immunoreactivity against mouse serum albumin (green) in the paraventricular area (PVA), primary somatosensory cortex (S1), the lateral hypothalamus (LH) and the ventral posteromedial nucleus of the thalamus (VPM), in a mouse that experienced acute high-dose radiation (AH), fractionated radiation corresponding to "Free moving conditioning" (FC) or no radiation (Con). **b** Mean pixel intensities of mouse serum albumin immunoreactivity were not different among three groups in all of the brain areas (n = 3 mice each; PVA, $F_{2,6} = 2.64$, $p = 0.151$; S1, $F_{2,6} = 0.15$, $p = 0.866$; LH, $F_{2,6} = 1.61$, $p = 0.275$; VPM, $F_{2,6} = 0.85$, $p = 0.472$; one-way ANOVA). Pixel intensities of the 8-bit images were quantified as an absolute value (abs., 0-255) using ImageJ. Open circles indicate individual data. Values are mean ± SEM. Source data are provided as a Source Data file.

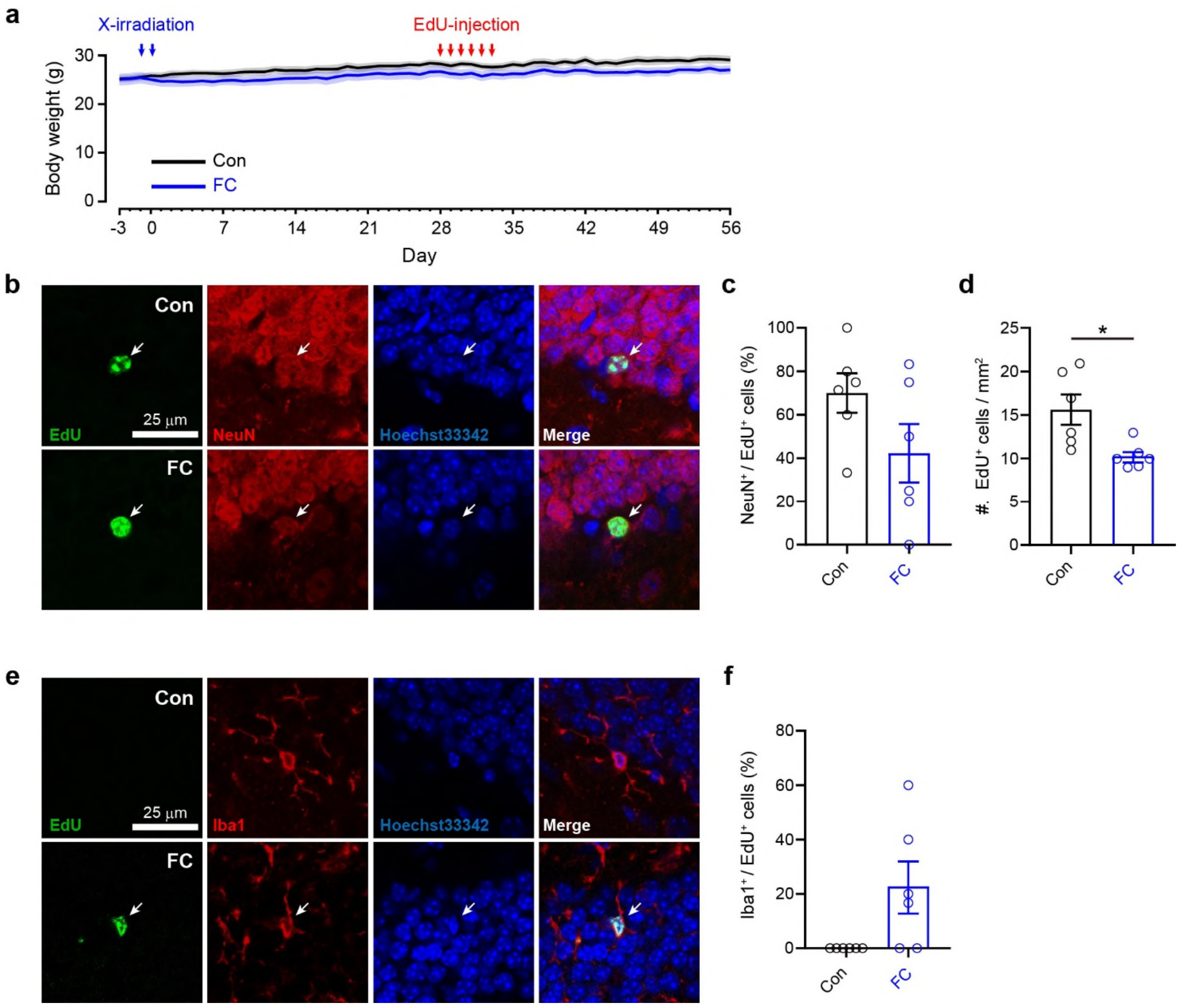

Supplementary Fig. 14 Body weight and neurogenesis in the hippocampal dentate gyrus after X-irradiation. a Body weight of the mice that experienced X-irradiation (blue arrows) with the "Free moving conditioning" protocol (FC; blue, *n* = 6 mice) or no radiation (Con; black, *n* = 6 mice). Red arrows indicate the timings of EdU (10 mg/ml) injection. Thick line: mean, Shadows: ± SEM. No significant difference was observed between two groups ($F_{1,10} = 2.27$, $p = 0.163$, two-way ANOVA). All mice survived for 56 days after radiation. **b** Confocal images showing EdU-positive cells co-expressing NeuN (arrows) at the subgranular zone of the hippocampal dentate gyrus in the X-irradiated (FC, bottom) or the control (Con, top) mice. The mice were perfused at 28 days after the first day of EdU injection. **c** Quantification of NeuN-positive cells among EdU-positive cells in the subgranular zone (*n* = 6 mice each, $p = 0.193$, Mann-Whitney U test, two-sided). **d** Quantification of EdU-positive cells indicates that the M.C. radiation impaired stem cell proliferation (*n* = 6 mice each). *$p = 0.0108$, Mann-Whitney U test, two-sided. **e** Confocal images of Iba1-positive cells at the dentate gyrus of the X-irradiated (FC, bottom) or non-X-irradiated (Con, top) mice. **f** In 4 out of 6 X-irradiated mice, Iba1-positive cells were found among EdU-positive cells, whereas no such newly generated microglial cells were found in the control mice (*n* = 6 mice each). $p = 0.0606$, Mann-Whitney U test, two-sided. Open circles indicate individual data. Values are mean ± SEM. Source data are provided as a Source Data file.

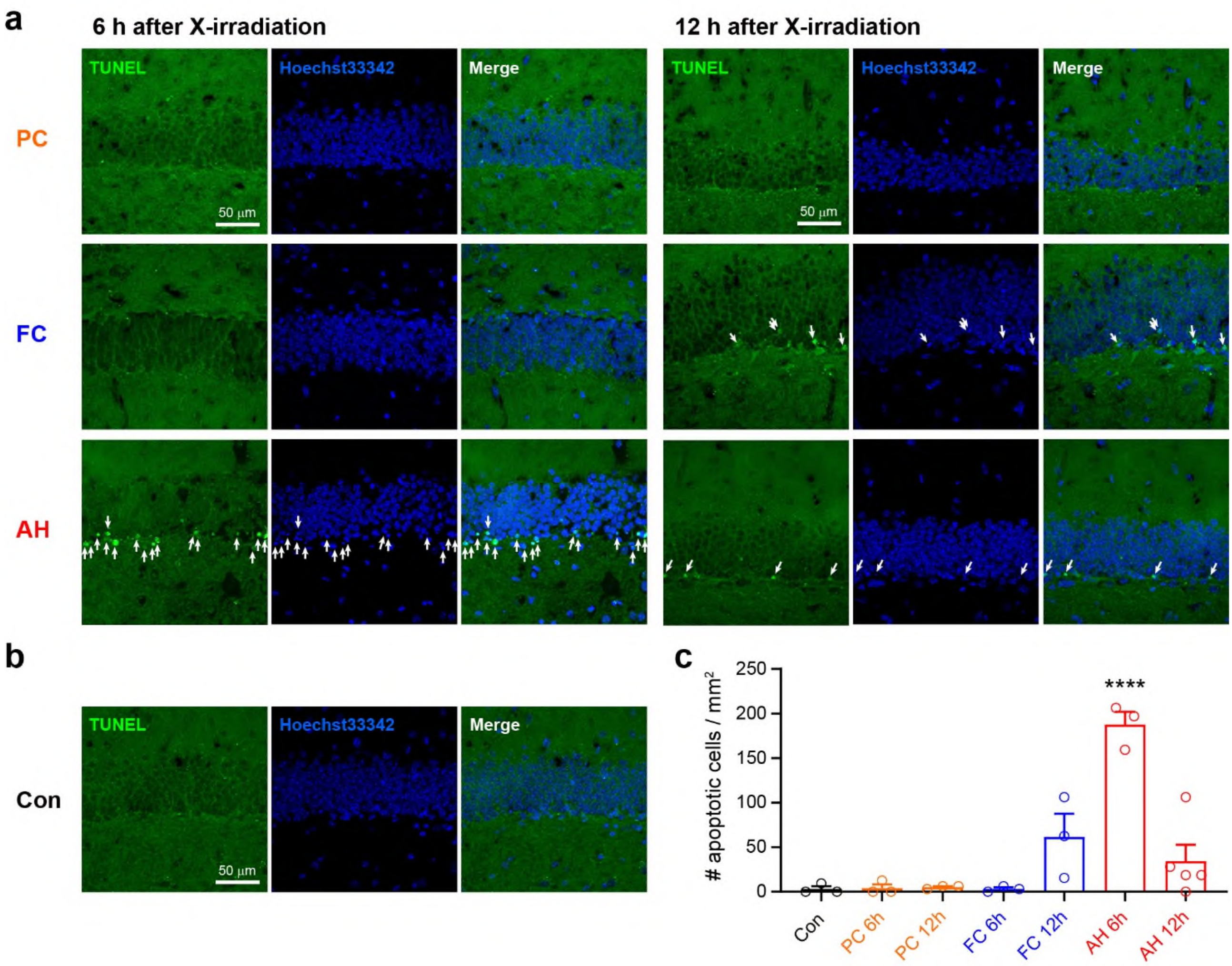

Supplementary Fig. 15 Apoptotic signals in the hippocampal dentate gyrus after X-irradiation. **a** Confocal images of TUNEL staining in the hippocampal slices obtained from the mice irradiated with X-rays. The mice were perfused 6 (left) or 12 (right) h after the last fraction of X-irradiation. PC: "Pulsed conditioning", FC: "Free moving conditioning", AH: Acute high-dose radiation. Arrows indicate apoptotic cells. **b** TUNEL staining in the non-X-irradiated control (Con) mice. **c** Quantification of the number of apoptotic cells in the hippocampal dentate gyrus (Con, n = 3 mice; PC 6 h, n = 3 mice; PC 12h, n = 3 mice; FC 6 h, n = 3 mice; FC 12 h, n = 3 mice; AH 6 h, n = 3 mice; AH 12 h, n = 5 mice). Con vs. PC 6h, $p > 0.9999$; Con vs. PC 12h, $p = 0.999$; Con vs. FC 6h, $p > 0.9999$; Con vs. FC 12h, $p = 0.0867$; Con vs. AH 6h, ****$p < 0.0001$; Con vs. AF 12h, $p = 0.463$; Dunnett's multiple comparisons test, two-sided. Open circles indicate individual data. Values are mean ± SEM**.** Source data are provided as a Source Data file.

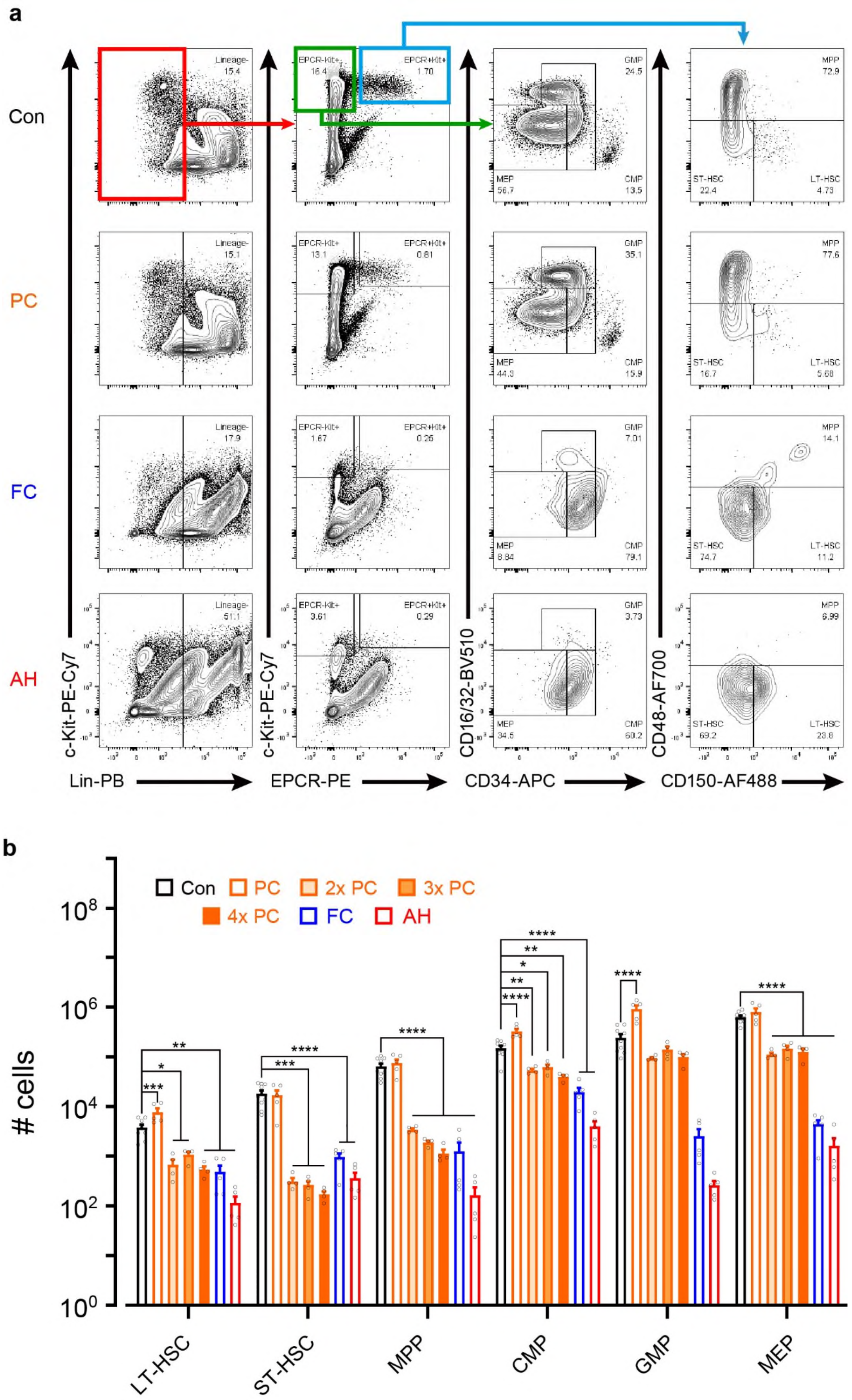
a
Con
PC
FC
AH
c-Kit-PE-Cy7
Lin-PB
EPCR-PE
CD16/32-BV510
CD34-APC
CD48-AF700
CD150-AF488
Lineage-
EPCR-Kit+
EPCR+Kit+
GMP
MEP
CMP
MPP
ST-HSC
LT-HSC
b
cells
Con
PC
2x PC
3x PC
4x PC
FC
AH
LT-HSC
ST-HSC
MPP
CMP
GMP
MEP

Supplementary Fig. 16 FACS analysis of the bone marrow cells in X-irradiated mice. a Representative FACS plots of the bone marrow obtained at 3 days after the last fraction of X-irradiation. Con: no radiation control, PC: "Pulsed conditioning", FC: "Free moving conditioning", AH: acute high-dose radiation. **b** Absolute cell numbers of various cell populations in the bone marrow. 2x PC: double X-dose of PC, 3x PC: triple X-dose of PC, 4x PC: quadruple X-dose of PC, LT-HSC: long-term hematopoietic stem cell, ST-HSC: short-term hematopoietic stem cell, MPP: multipotent progenitor, CMP: common myeloid progenitor, GMP: granulocyte-myeloid progenitor, MEP: megakaryocyte-erythroid progenitor. Con, $n = 9$ mice; PC, $n = 5$ mice; 2x PC; $n = 4$ mice; 3x PC, $n = 4$ mice; 4x PC, $n = 4$ mice; FC, $n = 5$ mice; AH, $n = 5$ mice. LT-HSC: PC, ***$p = 0.0006$; 2x PC, *$p = 0.0118$; 3x PC, *$p = 0.0339$; 4x PC, **$p = 0.0081$; FC, **$p = 0.0034$; AH, **$p = 0.0011$; Dunnett's multiple comparison tests vs. Con, two-sided. ST-HSC: PC, $p = 0.998$; 2x PC, ***$p = 0.0001$; 3x PC, ***$p = 0.0001$; 4x PC, ***$p = 0.0001$; FC, ****$p < 0.0001$; AH, ****$p < 0.0001$; Dunnett's multiple comparison tests vs. Con, two-sided. MPP: PC, $p = 0.811$; 2x PC, ****$p < 0.0001$; 3x PC, ****$p < 0.0001$; 4x PC, ****$p < 0.0001$; FC, ****$p < 0.0001$; AH, ****$p < 0.0001$; Dunnett's multiple comparison tests vs. Con, two-sided. CMP: PC, ****$p < 0.0001$; 2x PC, **$p = 0.0059$; 3x PC, *$p = 0.0130$; 4x PC, **$p = 0.0015$; FC, ****$p < 0.0001$; AH, ****$p < 0.0001$; Dunnett's multiple comparison tests vs. Con, two-sided. GMP: PC, ****$p < 0.0001$; 2x PC, $p = 0.509$; 3x PC, $p = 0.819$; 4x PC, $p = 0.550$; FC, $p = 0.0630$; AH, $p = 0.0595$; Dunnett's multiple comparison tests vs. Con, two-sided. MEP: PC, $p = 0.258$; 2x PC, ****$p < 0.0001$; 3x PC, ****$p < 0.0001$; 4x PC, ****$p < 0.0001$; FC, ****$p < 0.0001$; AH, ****$p < 0.0001$; Dunnett's multiple comparison tests vs. Con, two-sided. N.S. indicate no significant differences vs. the control. Open circles indicate individual data. Values are mean ± SEM. Source data are provided as a Source Data file.

Supplementary Table 1. PCR primers used in this study.

Gene Name	Forward Primer Sequence (5'–>3')	Reverse Primer Sequence (5'–>3')
Cre	TCTGGCATTTCTGGGGATTG	GTGCTAACCAGCGTTTTCGTTC
