# Supplementary material for: Remote control of neural function by X-ray-induced scintillation
Source: Nat Commun. 2021 Jul 22;12:4478. doi: 10.1038/s41467-021-24717-1 (PMC8298491; doi:10.1038/s41467-021-24717-1)
Supplement: Supplementary file 3 — Reporting Summary [file 41467_2021_24717_MOESM3_ESM.pdf]

nature portfolio

Corresponding author(s): Takayuki Yamashita
Last updated by author(s): Jun 17, 2021

Reporting Summary

Nature Portfolio wishes to improve the reproducibility of the work that we publish. This form provides structure for consistency and transparency in reporting. For further information on Nature Portfolio policies, see our Editorial Policies and the Editorial Policy Checklist.

Statistics

For all statistical analyses, confirm that the following items are present in the figure legend, table legend, main text, or Methods section.

n/a	Confirmed	
☐	☒	The exact sample size (n) for each experimental group/condition, given as a discrete number and unit of measurement
☐	☒	A statement on whether measurements were taken from distinct samples or whether the same sample was measured repeatedly
☐	☒	The statistical test(s) used AND whether they are one- or two-sided<br>*Only common tests should be described solely by name; describe more complex techniques in the Methods section.*
☒	☐	A description of all covariates tested
☐	☒	A description of any assumptions or corrections, such as tests of normality and adjustment for multiple comparisons
☐	☒	A full description of the statistical parameters including central tendency (e.g. means) or other basic estimates (e.g. regression coefficient) AND variation (e.g. standard deviation) or associated estimates of uncertainty (e.g. confidence intervals)
☐	☒	For null hypothesis testing, the test statistic (e.g. F, t, r) with confidence intervals, effect sizes, degrees of freedom and P value noted<br>*Give P values as exact values whenever suitable.*
☒	☐	For Bayesian analysis, information on the choice of priors and Markov chain Monte Carlo settings
☒	☐	For hierarchical and complex designs, identification of the appropriate level for tests and full reporting of outcomes
☒	☐	Estimates of effect sizes (e.g. Cohen's d, Pearson's r), indicating how they were calculated

Our web collection on statistics for biologists contains articles on many of the points above.

Software and code

Policy information about availability of computer code

Data collection	SutterPatch running on Igor Pro 8 (electrophysiology), BZ-X Viewer 01.01.01 (epifluorescence imaging), Zeiss ZEN 2.3 SP1(confocal imaging), PicoScope 6 (photosensor measurement), Amcap 9 and Wondershare Filmora scrn Version 2.0.1 (behavioral filming)
Data analysis	GraphPad Prism 8, Igor Pro 7, Igor Pro 8, ImageJ 1.53f, Matlab 2019a, DeepLabCut v2.1, Python 3, FlowJo 10.5.3.

For manuscripts utilizing custom algorithms or software that are central to the research but not yet described in published literature, software must be made available to editors and reviewers. We strongly encourage code deposition in a community repository (e.g. GitHub). See the Nature Portfolio guidelines for submitting code & software for further information.

Data

Policy information about availability of data

All manuscripts must include a data availability statement. This statement should provide the following information, where applicable:

- Accession codes, unique identifiers, or web links for publicly available datasets
- A description of any restrictions on data availability
- For clinical datasets or third party data, please ensure that the statement adheres to our policy

The data used to generate figures that support the findings of this study are freely available in the Open Access CERN database Zenodo: https://zenodo.org/communities/ty-lab-data with doi hyperlink: https://doi.org/10.5281/zenodo.4964867. Source data are provided with this paper.

Field-specific reporting

Please select the one below that is the best fit for your research. If you are not sure, read the appropriate sections before making your selection.

- [x] Life sciences
- [ ] Behavioural & social sciences
- [ ] Ecological, evolutionary & environmental sciences

For a reference copy of the document with all sections, see nature.com/documents/nr-reporting-summary-flat.pdf

Life sciences study design

All studies must disclose on these points even when the disclosure is negative.

Sample size	No statistical methods were used to determine sample size a priori. Sample size was determined based on the literature in the field.<br>[1] Chen, S. et al. Near-infrared deep brain stimulation via upconversion nanoparticle-mediated optogenetics. Science 359, 679!684 (2018).<br>[2] Kim, T.I. et al. Injectable, cellular-scale optoelectronics with applications for wireless optogenetics. Science 340, 211!216 (2013).<br>[3] Mathis, J. et al. Principles for applying optogenetic tools derived from direct comparative analysis of microbial opsins. Nat. Methods 9, 159–172 (2012).<br>[4] Tsai, H.C. et al. Phasic firing in dopaminergic neurons is sufficient for behavioral conditioning. Science 324, 1080–1084 (2009).<br>[5] Monje, M.L., Mizumatsu, S., Fike, J.R. & Palmer T.D. Irradiation induces neural precursor-cell dysfunction. Nat. Med. 8, 955–962 (2002).
Data exclusions	For CPP tests (Fig. 5), two mice which showed a biased preference for one chamber at Day0 (>85 %, as a pre-established criterion based on preliminary experiments) were excluded from the analysis. Otherwise, no data were excluded from the analysis.
Replication	All attempts of replication were successful. For all experiments, replication was performed at least three times and showed comparable results. The results from these experiments were pooled and used for analysis.
Randomization	Cells and animals were randomly assigned to each experimental group.
Blinding	Blinding was not possible for most experiments as group allocation and treatment was administered by the researcher collecting the data. For CPP tests (Fig. 5), immunostaining against serum albumin (Supplementary Fig. 13) and DCX (Fig. 6), EdU staining (Supplementary Fig. 14b-f), and TUNEL assays (Supplementary Fig. 15), data were analyzed by investigators blinded to group allocation. Otherwise, data were analyzed without blinding as all samples were processed by the same operators. The analysis was performed objectively and not subjective to human bias.

Reporting for specific materials, systems and methods

We require information from authors about some types of materials, experimental systems and methods used in many studies. Here, indicate whether each material, system or method listed is relevant to your study. If you are not sure if a list item applies to your research, read the appropriate section before selecting a response.

Materials & experimental systems

n/a	Involved in the study	
☐	☒	Antibodies
☐	☒	Eukaryotic cell lines
☒	☐	Palaeontology and archaeology
☐	☒	Animals and other organisms
☒	☐	Human research participants
☒	☐	Clinical data
☒	☐	Dual use research of concern

Methods

n/a	Involved in the study	
☒	☐	ChIP-seq
☐	☒	Flow cytometry
☒	☐	MRI-based neuroimaging

Antibodies

Antibodies used	anti-tyrosine hydroxylase, rabbit polyclonal, 1:1000, Merck Millipore (catalog: AB152);<br>anti-Iba1, rabbit polyclonal, 1:500, Wako (catalog: 019-19741);<br>anti-GFAP, mouse monoclonal (clone: GA5), 1:1000, Merck Millipore (catalog: MAB360);<br>anti-NeuN, mouse monoclonal (clone: clone A60), 1:500, Merck Millipore (catalog: MAB377);<br>anti-mouse serum albumin, goat polyclonal, 1:1000, Abcam (catalog: ab19194);<br>anti-doublecortin, rabbit polyclonal, 1:1000, Abcam (catalog: ab18723);<br>anti-c-Fos, rabbit monoclonal (clone: EPR21930-238), 1:1000, Abcam (catalog: ab222699);<br>biotinylated anti-NK1.1, mouse monoclonal (clone: PK136), 1:500, Biolegend (catalog: 108704);<br>biotinylated anti-CD11b, rat monoclonal (clone: M1/70), 1:500, Biolegend (catalog: 101204);<br>biotinylated anti-Ter119, rat monoclonal (clone: Ter119), 1:500, Biolegend (catalog: 116204);<br>biotinylated anti-Gr-1, rat monoclonal (clone: RB6-8C5), 1:500, Biolegend (catalog: 108404);

biotinylated anti-CD4, rat monoclonal (clone: GK1.5), 1:500, Biolegend (catalog: 100404);
biotinylated anti-CD8α, rat monoclonal (clone: 53-6.7), 1:500, Biolegend (catalog: 100704);
biotinylated anti-CD3ε, Armenian hamster monoclonal (clone: 145-2C11), 1:500, Biolegend (catalog: 100304);
biotinylated anti-B220, monoclonal (clone: RA3-6B2), 1:500, Biolegend (catalog: 103204);
biotinylated anti-IL-7Rα, rat monoclonal (clone: SB/199), 1:500, Biolegend (catalog: 121104);
CF594-conjugated anti-rabbit IgG, donkey polyclonal, 1:1000, Biotium (catalog: 20152);
CF488A-conjugated anti-rabbit IgG, donkey polyclonal, 1:1000, Biotium (catalog: 20015);
CF488A-conjugated anti-mouse IgG, goat polyclonal, 1:1000, Biotium (catalog: 20018);
CF488A-conjugated anti-goat IgG, donkey polyclonal, 1:1000, Biotium (catalog: 20016);
BV510-conjugated anti-CD16/32, rat monoclonal (clone: 93, 1:50, Biolegend (catalog: 101333);
PE-Cy5-conjugated anti-CD135, rat monoclonal (clone: A2F10, 1:100, Biolegend (catalog: 135312);
AF700-conjugated anti-CD48, Armenian hamster monoclonal (clone: HM48-1), 1:100, Biolegend (catalog: 103426);
AF488-conjugated anti-CD150, rat monoclonal (clone: TC15-12F12.2), 1:100, Biolegend (catalog: 115916);
PE-conjugated anti-EPCR, rat monoclonal (clone: eBio1560, 1:100, Thermo Fisher Scientific (catalog: 12-2012082);
PE-Cy7-conjugated anti-c-Kit, rat monoclonal (clone: 2B8), 1:100, Biolegend (catalog: 105814);
APC-conjugated anti-CD34, Armenian hamster monoclonal (clone: HM34), 1:50, Biolegend (catalog: 128612)

Validation

Validation statements of commercial antibodies are available from manufacturers:
・ anti-tyrosine hydroxylase (https://www.merckmillipore.com/JP/ja/product/Anti-Tyrosine-Hydroxylase-Antibody,MM_NF-AB152#documentation);
・ anti-Iba1 (https://labchem-wako.fujifilm.com/jp/product_data/docs/00055446_doc01.pdf);
・ anti-GFAP (https://www.merckmillipore.com/JP/ja/product/Anti-Glial-Fibrillary-Acidic-Protein-Antibody-clone-GA5,MM_NF-MAB360#documentation);
・ anti-NeuN (https://www.merckmillipore.com/JP/ja/product/Anti-NeuN-Antibody-clone-A60,MM_NF-MAB377#documentation);
・ anti-mouse serum albumin (https://www.abcam.co.jp/mouse-serum-albumin-antibody-ab19194.html);
・ anti-doublecortin (https://www.abcam.co.jp/doublecortin-antibody-ab18723.html);
・ anti-c-Fos (https://www.abcam.co.jp/c-fos-antibody-epr21930-238-ab222699.html);
・ biotinylated anti-NK1.1 (https://www.biolegend.com/ja-jp/products/biotin-anti-mouse-nk-1-1-antibody-428);
・ biotinylated anti-CD11b (https://www.biolegend.com/ja-jp/products/biotin-anti-mouse-human-cd11b-antibody-346);
・ biotinylated anti-Ter119 (https://www.biolegend.com/ja-jp/products/biotin-anti-mouse-ter-119-erythroid-cells-antibody-1864);
・ biotinylated anti-Gr-1 (https://www.biolegend.com/ja-jp/products/biotin-anti-mouse-ly-6g-ly-6c-gr-1-antibody-457);
・ biotinylated anti-CD4d (https://www.biolegend.com/ja-jp/products/biotin-anti-mouse-cd4-antibody-247);
・ biotinylated anti-CD8α (https://www.biolegend.com/ja-jp/products/biotin-anti-mouse-cd8a-antibody-152);
・ biotinylated anti-CD3ε (https://www.biolegend.com/ja-jp/products/biotin-anti-mouse-cd3epsilon-antibody-22);
・ biotinylated anti-B220 (https://www.biolegend.com/ja-jp/products/biotin-anti-mouse-human-cd45r-b220-antibody-444);
・ biotinylated anti-IL-7Rα (https://www.biolegend.com/ja-jp/products/biotin-anti-mouse-cd127-il-7ralpha-antibody-3048);
・ CF594- or CF488A-conjugated anti-rabbit IgG (https://biotium.com/product/donkey-anti-rabbit-igg-hl-highly-cross-adsorbed/);
・ CF488A-conjugated anti-mouse IgG (https://biotium.com/product/goat-anti-mouse-igg-hl-highly-cross-absorbed/);
・ CF488A-conjugated anti-goat IgG (https://biotium.com/product/donkey-anti-goat-igg-hl-highly-cross-adsorbed/);
・ BV510-conjugated anti-CD16/32 (https://www.biolegend.com/ja-jp/products/brilliant-violet-510-anti-mouse-cd16-32-antibody-9917);
・ PE-Cy5-conjugated anti-CD135 (https://www.biolegend.com/ja-jp/products/pe-cyanine5-anti-mouse-cd135-antibody-6300);
・ AF700-conjugated anti-CD48 (https://www.biolegend.com/ja-jp/products/alexa-fluor-700-anti-mouse-cd48-antibody-6670);
・ AF488-conjugated anti-CD150 (https://www.biolegend.com/ja-jp/products/alexa-fluor-488-anti-mouse-cd150-slam-antibody-3328);
・ PE-conjugated anti-EPCR (https://www.thermofisher.com/antibody/product/CD201-EPCR-Antibody-clone-eBio1560-1560-Monoclonal/12-2012-82);
・ PE-Cy7-conjugated anti-c-Kit (https://www.biolegend.com/ja-jp/products/pe-cyanine7-anti-mouse-cd117-c-kit-antibody-1900);
・ APC-conjugated anti-CD34 (https://www.biolegend.com/ja-jp/products/apc-anti-mouse-cd34-antibody-6520);

We also validated the specificity of the antibodies for immunostaining (anti- tyrosine hydroxylase, anti-Iba1, anti-GFAP, anti-NeuN, anti-mouse serum albumin, anti-doublecortin, anti-c-Fos, CF594- or CF488A-conjugated anti-rabbit IgG, CF488A-conjugated anti-mouse IgG, and CF488A-conjugated anti-goat IgG) on fixed mouse coronal slices and confirmed by comparing immunofluorescence signal with literature listed in the websites. Staining with anti-mouse serum albumin (shown in supplementary fig. 13) was further confirmed by staining injured mouse brains as a positive control.

Eukaryotic cell lines

Policy information about cell lines

Cell line source(s)

HEK293 (AAV-293) cell line bought from Agilent (catalog: 240073) was used for AAV production. HEK293 cell line purchased from JCRB Cell Bank (catalog:JCRB 9068) was used for Glosensor experiments. HEK293 cell line kindly provided by Dr. Makoto Tominaga (National Institute for Physiological Sciences, Okazaki, Japan) was used for electrophysiology. The HEK 293 cell line provided by Dr. Makoto Tominaga was originally gifted by Dr. David Julius at the University of California, San Francisco. This cell line has been used for many studies in their laboratory (ex. Tominaga et al., Neuron 21: 531-543, 1998) and is not commercially available.

Authentication

We authenticated HEK293 cell lines based on the morphology under microscope and the analysis of the growth curve.

Mycoplasma contamination	No mycloplasma contamination
Commonly misidentified lines (See ICLAC register)	No commonly misidentified lines were used.

Animals and other organisms

Policy information about studies involving animals; ARRIVE guidelines recommended for reporting animal research

Laboratory animals	C57BL6/J mice (8-18 week-old, both sexes); homozygous offspring (8-23 week-old, both sexes) of DAT-IRES-Cre mice
Wild animals	No wild animals were used in this study.
Field-collected samples	No field samples were used in this study.
Ethics oversight	All experiments were performed in accordance with the guidelines of the Physiological Society of Japan and approved by the institutional review board of the Research Institute of Environmental Medicine, Nagoya University, Japan or by the Institutional Animal Care and Use Committee of Fujita Health University, Japan.

Note that full information on the approval of the study protocol must also be provided in the manuscript.

Flow Cytometry

Plots

Confirm that:

- [x] The axis labels state the marker and fluorochrome used (e.g. CD4-FITC).
- [x] The axis scales are clearly visible. Include numbers along axes only for bottom left plot of group (a 'group' is an analysis of identical markers).
- [x] All plots are contour plots with outliers or pseudocolor plots.
- [x] A numerical value for number of cells or percentage (with statistics) is provided.

Methodology

Sample preparation	Two long bones (femur and tibia) were sampled per mouse and bone marrow (BM) cells were flushed out with a syringe containing 10 ml of FACS buffer and a 25G needle to create a single-cell suspension. Cells were treated with ACK buffer to lyse red blood cells (1 minute) and stained with fluorescently conjugated antibodies for 30 minutes, shielded from light. All procedures were performed on ice. Cells were resuspended in FACS buffer containing Hoechst for viability staining and filtered before analysis.
Instrument	FACSAria III Cell Sorter (Beckton, Dickinson and Company)
Software	FlowJo 10.5.3.
Cell population abundance	Sorting was not performed in this study.
Gating strategy	FSC-A/SSC-A (x-axis/y-axis) for BM lymphocytes, FSC-H/FSC-W followed by SSC-H/SSC-W to gate for singlets, Hoechst and lineage cocktail for live Lin- cells, EPCR/c-Kit double-positive cells (EPCR+c-Kit+) followed by SLAM markers CD150/CD48 to gate for MPPs (CD150+/-CD48+), ST-HSCs (CD150-CD48-) and LT-HSCs (CD150+CD48-). For mature cell fractions, cells were gated with EPCR-c-Kit+ followed by CD34/CD16/32 for GMPs (CD34+CD16/32+Lin-c-Kit+), MEPs (CD34-CD16/32lowLin-c-Kit+) and CMPs (CD34+CD16/32lowLin-c-Kit+). Refer to Supplementary Figure 16a for details. Unstained versus single stains were used to determine boundaries of positive and negative populations (cells above 103 were generally considered positive) together with contour plots as additional guides. The gating strategy fixed for the control condition was applied to all other samples.

- [x] Tick this box to confirm that a figure exemplifying the gating strategy is provided in the Supplementary Information.
