# Supplementary material for: Remote control of neural function by X-ray-induced scintillation
Source: Nat Commun. 2021 Jul 22;12:4478. doi: 10.1038/s41467-021-24717-1 (PMC8298491; doi:10.1038/s41467-021-24717-1)
Supplement: Supplementary file 2 — Peer Review File [file 41467_2021_24717_MOESM2_ESM.pdf]

Editorial Note: This manuscript has been previously reviewed at another journal that is not operating a transparent peer review scheme. This document only contains reviewer comments and rebuttal letters for versions considered at *Nature Communications*.

REVIEWERS' COMMENTS

Reviewer #1 (Remarks to the Author):

The authors have sufficiently addressed my comments.

Reviewer #2 (Remarks to the Author):

My concerns have been addressed in the previous revisions. I have no further concerns.

Response to reviewers

Reviewer #1 *(reviewer's comments in italic)***:**
The authors have sufficiently addressed my comments.

We thank the reviewer for his/her constructive comments to improve the manuscript.

Reviewer #2 *(reviewer's comments in italic)***:**
My concerns have been addressed in the previous revisions. I have no further concerns.

We thank the reviewer for his/her constructive comments to improve the manuscript.
